# Mechanochemistry-assisted hydrolysis of softwood over stable sulfonated carbon catalysts in a semi-batch process†

**DOI:** 10.1039/c9ra07668a

**Published:** 2019-10-18

**Authors:** David Scholz, Jingwei Xie, Oliver Kröcher, Frédéric Vogel

**Affiliations:** Paul Scherrer Institute 5232 Villigen PSI Switzerland frederic.vogel@psi.ch; École Polytechnique Fédérale de Lausanne 1015 Lausanne Switzerland; Georgia Institute of Technology Atlanta GA 30332 USA; Fachhochschule Nordwestschweiz 5210 Windisch Switzerland

## Abstract

The hydrolysis of lignocellulose is the first step in saccharide based bio-refining. The recovery of homogeneous acid catalysts imposes great challenges to the feasibility of conventional hydrolysis processes. Herein, we report a strategy to overcome these limitations by using stable sulfonated carbons as solid acid catalysts in a two-step process, composed of mechanocatalytic pretreatment and secondary hydrolysis in a semi-batch reactor. Without mechanocatalytic pre-treatment the hydrolysis of the insoluble substrate largely occurs through homogeneously catalyzed reactions. Ball-milling induced amorphization promotes a substantially higher substrate reactivity, because homogeneous hydrolysis occurs preferentially from less ordered structural domains in cellulose. In contrast, concerted ball-milling (CBM) of cellulose with the sulfonated carbon promotes a heterogeneously catalyzed hydrolysis to soluble oligosaccharides. By performing an in-depth physicochemical characterization of cellulose subjected to CBM treatment with different carbons, we reveal the crucial role of strong Brønsted acid sites in facilitating mechanocatalytic depolymerization. Recyclability experiments confirmed that despite being subject to profound structural changes during repeated pre-treatment/semi-batch hydrolysis cycles, the sulfonated carbon retained its catalytic activity. The combination of mechanocatalytic pretreatment with strong solid acids and hydrolysis in the semi-batch reactor was successfully extrapolated for the first time to the hydrolysis of real lignocellulose to achieve quantitative yields in C_5_ and high yields in C_6_ derived products.

## Abstract 2

A two-step process employing stable sulfonated carbons, overcomes the challenging recyclability of mineral acids used in conventional hydrolysis processes.

## Graphical abstract

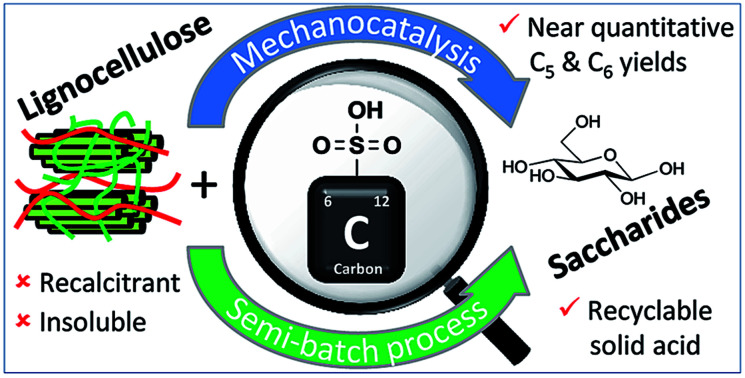

## Introduction

Owing to its widespread abundance, lignocellulosic biomass is regarded as a promising substitute to replace fossil-resources as a feedstock for the production of fuels and chemicals.^1^ Cellulose (40–55%),^2^ the most abundant component of lignocellulose, is a syndiotactic homopolymer consisting of β-d-glucopyranose units connected *via* β-1,4-glycosidic bonds. The polymer chains are held together by an extended intra- and inter-chain hydrogen bonding network that facilitates the occurrence of chemically recalcitrant crystalline domains.^3^

Chemical valorisation of cellulose can be accomplished by hydrolytic cleavage of the glycosidic bond. Different approaches based on enzyme catalysts,^4^ thermochemical processing^5^ and Brønsted acid catalysts^6^ have been studied. Hydrolysis processes relying on the action of enzymes yield monosaccharides with high selectivity at mild reaction conditions, but suffer from slow reaction rates, limited catalyst stability and high costs for the catalysts.^7^ Even in the absence of catalysts, cellulose undergoes auto-hydrolysis in hot compressed water at temperatures around 230–240 °C.^8^ In this case, the protons required for hydrolysis are provided by the self-ionization equilibrium of water which reaches a maximum at approximately 300 °C.^9^ Hydrolysis can also be performed at milder conditions through the use of homogeneous Brønsted acid catalysts. During the 20^th^ century different attempts were made to implement hydrolysis processes for the production of monosaccharides from lignocellulose, using concentrated^10,11^ or diluted^12–14^ mineral acid catalysts. Although some of these processes have yielded saccharides with impressive yields, none of them have so far been commercialized due to unfavourable process economics resulting from the recovery or the neutralization of the homogeneous acid.^14^ To this end, the use of solid acid catalysts would offer clear advantages. Heterogeneous catalysts can be more easily recycled and are less prone to induce corrosion in reactor materials.^15^ Nonetheless, the use of solid acid catalysts also imposes new challenges.

Firstly, the metal-oxide,^16,17^ zeolite^17,18^ or polymer^19,20^ based-materials that have been tested in this application, all lack long-term stability under the hydrothermal conditions typical of lignocellulose hydrolysis processes.^21–24^ Substantial progress in this regard has been made in recent years through the development of acid functionalized carbons. Carbon materials display higher stability in the liquid phase because polyaromatic systems do not undergo hydrolytic attack,^25^ which can lead to dissolution. Different oxidation^26,27^ and sulfonation^28^ strategies have been developed to introduce carboxylic or sulfonic acid surface functionalities into carbons. Both of these display activity in cellulose conversion, however, hydrolysis with carboxylic acid functionalized materials typically requires higher temperatures (180–230 °C)^26,29–32^ to proceed at acceptable rates as compared to materials containing sulfonic acid groups (100–150 °C).^17,33–36^ This observation can be rationalized with the fact that hydrolysis proceeds *via* protonation of the weakly basic glycosidic oxygen which has been shown to be favoured by strong Brønsted acids (p*K*_a_ ≤ −3).^37^

Another marked difference between carboxylic and sulfonic acid group functionalized materials is, that the latter was believed to be less stable due to the propensity of sulfonic acid groups to leach in hydrothermal media and to undergo deactivation *via* ion exchange induced proton leaching.^38^ However, we recently demonstrated that stable sulfonated carbons can be prepared by exploiting the dependence of sulfonic acid group stability on the structure of the carbonaceous support.^39^ The stability of sulfonic acid groups in carbons is directly linked to their proximity to activating/deactivating substituents that increase/decrease the tendency of the C–S bond to undergo solvolysis. Furthermore, their deactivation *via* proton leaching can be fully overcome by the addition of complexation agents to the reaction medium which suppress the ability of cations to participate in ion exchange processes.^39^ Both modes of deactivation are particularly relevant for biomass conversion processes as these are typically performed in hydrothermal media and because most biomass feedstocks contain 0.3–1.0% of minerals.^40^

A second challenge is related to the constraints imposed on the reaction system by the insolubility of cellulose. The insolubility should be anticipated to strongly impede a heterogeneously catalysed hydrolysis. Although mechanisms involving a collision of particles in suspension^29,30,41^ or adsorption^35,36^ of cello-oligosaccharides have been proposed, neither of these have been underlined by experimental results. Nonetheless, several previous studies have reported that good yields (50.4–74.5%) can be achieved during the hydrolysis of pristine^35,42^ or mechanically activated^18^ (ball-milled) cellulose with solid acids. It however remains uncertain to what degree the reaction truly occurred over heterogenized acid sites and to what extent homogeneously catalysed reactions (*i.e.* auto-hydrolysis or hydrolysis by leached species) contributed to product formation.

A strategy proposed to overcome the mass-transfer limitations is mechanocatalytic depolymerization, which can be achieved by the concerted ball-milling (CBM) of a solid acid with cellulose.^43^ When mechanocatalytic depolymerization is performed with homogeneous catalysts,^44^ the rate of depolymerization has been demonstrated to exhibit a similar dependence on the p*K*_a_ of the employed acid, as found for the aqueous phase hydrolysis.^37^ The characteristics of effective homogeneous catalysts should also apply to solid acid catalysts, that is to say, materials featuring a high density of strong Brønsted acid sites should display higher activity during mechanocatalytic depolymerization. This hypothesis is supported by previous studies that have investigated the efficacy of various solid acids during CBM treatment. Kobayashi *et al.* showed that CBM treatment with a carboxylic acid functionalized carbon does not induce mechanocatalytic depolymerization but enhances the propensity of cellulose to undergo hydrolysis.^29^ The CBM treated substrate (substrate/catalyst (S/C) = 6.5, 60 rpm, 48 h) could be hydrolysed at 180 °C to yield 21.3% and 70.0% mono- and oligosaccharides, respectively, as opposed to 3.8% and 10.0% when individually ball-milled cellulose was converted under the same conditions.^29^ In this study, a higher selectivity to monosaccharides could only be achieved when a secondary aqueous phase hydrolysis was performed in presence of strong homogeneous Brønsted acids. When CBM is performed with solid acids featuring strong Brønsted acid sites, cellulose undergoes extensive mechanocatalytic depolymerization. Mechanocatalysis with metal-oxides (niobium molybdate^45^ or kaolinite^43^) or resins (Amberlyst-70 (ref. 30) and Nafion^46^) has been shown to afford soluble oligosaccharides at 72.0–99.0% yield (S/C = 1–6.5, 60–800 rpm, 3–48 h). Although these results indicate the principal feasibility of this approach for overcoming the restrained substrate–catalyst contact, it has also shown that metal-oxides^47^ and Amberlyst^30^ are not recyclable after the CBM treatment due to structural degradation and deactivation of the materials. Consequently, so far, no catalytic processes have been developed that employ mechanocatalytic pre-treatment with strong solid acid catalysts. Furthermore, previous studies have focused on studying the mechanocatalytic formation of oligosaccharides from model substrates rather than real lignocellulosic biomass.

The issues addressed before demonstrate the beneficial effect of employing strong Brønsted acid catalysts for the hydrolysis of cellulose and lignocellulose. The lacking understanding of the mechanism of this reaction and the unavailability of stable catalysts has so far hampered the ability to design continuous-flow catalytic processes for the conversion of real lignocellulosic biomass. Therefore, in this study, we develop a semi-batch hydrolysis process using a hydrothermally and mechanically stable sulfonated carbon as a strong solid acid catalyst. We shed light on the crucial role of homogeneously catalysed reactions in this application and reveal the structural features of cellulose that dictate its reactivity. In a second step, we investigate the efficacy of the sulfonated carbon catalyst in facilitating a mechanocatalytic depolymerization and identify the active sites over which the reaction occurs. By demonstrating the high stability of the sulfonated carbon catalyst we form the basis for designing a two-step catalytic process, in which mechanocatalytically pretreated cellulose is hydrolysed in a semi-batch reactor. We extrapolate this methodology for the first time to the conversion of real lignocellulose and show that the obtained yields are competitive with those achieved with state-of-the-art homogeneous acid based processes.

## Experimental

### Materials

d-(+)-cellobiose (Sigma-Aldrich, ≥98%), d-(+)-glucose mono-hydrate (Sigma-Aldrich, 99%), d-(+)-fructose (Fluka, >99%), d-(+)-mannose (Sigma-Aldrich, 99%), d-(+)-galactose (Sigma-Aldrich, 99%), 5-HMF (Sigma-Aldrich, 99%), 1,6-anhydro-β-d-glucose (Sigma-Aldrich, 99%), levulinic acid (Sigma-Aldrich, 99%), formic acid (Sigma-Aldrich, 98%), d-(+)-xylose (Sigma-Aldrich, ≥99%), l-(+)-arabinose (Sigma-Aldrich, ≥99%) and furfural (Sigma-Aldrich, 99%) were used as external calibration standards.

### Analysis

Analyte quantification was achieved by means of an HPLC system (Agilent 1260 Infinity) equipped with an Aminex Biorad monosaccharide analysis column (HPX-87C, 300 × 7.8 mm) and a refractive index detector. The column was operated at 80 °C and with a flowrate of 0.45 mL min^−1^ using Millipore water as the eluent.

The conversion (*X*) of cellulose (Sigma-Aldrich, Avicel PH-101) was calculated by gravimetric analysis according to the following equation, *X* = 1 − (*m*_s_ − *m*_cat_)/*m*_Cel_. *m*_s_ denotes the dry mass of solids recovered after an experiment and *m*_cat_ and *m*_Cel_ the catalyst and cellulose mass charged into the reactor, respectively. The yield of reaction product *i* (*Y*_*i*_) was calculated by using the following formula, *Y*_*i*_ = *c*_*i*_*V*_p_/*n*_Gluc._ in which *c*_*i*_ denotes the concentration of reaction product *i*, *V*_p_ the total volume of reaction products and *n*_Gluc._ the moles of glucose bound in cellulose (calculated by assuming a molecular mass of *M*_w_ = 162 g mol^−1^ for cellulose-bound glucose). The selectivity (*S*_*i*_) in formation of reaction product *i* was calculated using the following formula, *S*_*i*_ = *Y*_*i*_/*X*. Oligosaccharides were quantified by post-hydrolysis according to the method described by NREL.^48^ Identification of unknown volatile substances was accomplished with an Agilent 5977A Series GC/MSD equipped with a quadrupole MS detector and a HP-5MS UI column. Oligosaccharides were identified by measuring the ESI total-ion chromatograms on a Thermo Scientific Ultimate 3000 equipped with a QExactive Focus mass spectrometer.

### Catalyst synthesis and characterization

The detailed procedures for preparation and characterization of the sulfonated carbon catalyst are described elsewhere.^39^ Deactivation of the as prepared catalyst was achieved through three consecutive ion exchange treatments (1 g per 15 mL solution, 30 °C, 16.5 h) using an aqueous solution containing 0.25 wt% of each Na^+^, K^+^, Ca^2+^ and Mg^2+^.

### Semi-batch reactor

The hydrolysis of cellulose and softwood was performed in a stirred high pressure reactor vessel (Parr Instrument Company HP/HT 4576A, 250 mL) connected at the inlet to an HPLC pump (Knauer P4.1S) and at the outlet to an in-house made heat-exchanger. The pressure (20 bar) over the system was maintained by a back pressure valve (Swagelok KHB1W0A4C2P60000) installed in series with the heat-exchanger. The reactor effluent was filtered through a 500 nm porous metal-frit (Mottcorp 4001130–005). During a typical experiment the sulfonated carbon catalyst (2 g), the substrate (7 g) and the eluent were charged into the reactor and the flowrate of the HPLC pump was set to 7 mL min^−1^. After the system was pressurized the reaction was initialized, by heating to 165 °C. The reactor effluent was collected in a Schott bottle, which was placed on a balance (Sartorius LC6201S) to monitor the effluent flow-rate. The reaction progress was monitored by taking samples in regular intervals at the outlet of the back-pressure valve or directly from the effluent collection bottle. After a 6 h experiment, the flow-rate of the HPLC pump was reduced to 2 mL min^−1^ and the reaction was quenched by cooling the reactor with pressurized air for 0.5 h.

### Ball-milling and concerted ball-milling

Mechanical and mechanocatalytic pre-treatment of cellulose was performed in a sialon pot (80 mL) with sialon spheres (25 pcs. with 1 cm diameter) at 300 rpm by using a Fritsch Pulverisette 6. The total mass of cellulose and catalyst charged into the ball-mill was kept constant for all pre-treatments (9 g). The temperature in the ball-mill was monitored in regular intervals to exclude that a temperature induced degradation of the sample occurred.

### Characterization of pretreated cellulose

*Particle size*: The primary particle size distribution was measured on a Horiba LA-950V2 laser diffraction particle size analyser. *Powder XRD*: Powder XRD patterns of cellullose were measured on a D8 ADVANCE (Bruker) diffractometer over the range 2*θ* = 5–70° using Cu Kα radiation (*λ* = 0.154 nm). Deconvolution was performed on baseline-corrected XRD patterns by assuming Gaussian peak shape for all diffraction peaks.^49^ A Levenberg–Marquardt minimization algorithm optimized the peak position, width and area such as to achieve an optimal fit to the diffraction patterns (*R*^2^ ≥ 0.994 for all deconvoluted diffraction patterns). The crystallite size was calculated by using a shape factor of 0.94 in the Scherrer equation.^50^*FTIR spectroscopy*: FTIR spectra were recorded in the range 400–4000 cm^−1^ with a resolution of 2 cm^−1^ by using a Bruker Vertex 80 V FTIR spectrometer. FTIR spectra of the cellulose samples were recorded by preparing 1 wt% KBr pellets. Prior to preparing the pellets, cellulose samples and KBr were dried overnight in a convection oven set to 110 °C. *Elemental analysis*: The carbon, sulphur, nitrogen and hydrogen content of the different samples were measured by using an Elementar Vario EL Device. *SEM-EDX*: Electron microscopy and energy dispersive X-ray spectroscopy was performed on a Zeiss Ultra 55 field-emission-gun scanning electron microscope. To reduce charging effects, all samples were applied on conductive carbon tape and subsequently sputter coated (48 mA, 90 s) by using a Leica EM SCD 500 and an Au : Pd alloy (80 : 20) or chromium for EDX mapping. Images were acquired using an acceleration voltage of 7.0 kV and a magnification of 100, 500 and 2500×. *Solubility of cellulose samples*: The solubility of pre-dried samples (2 h, 110 °C) was measured by suspending cellulose in Millipore water (1 g per 10 mL, 25 °C, 1 h) under continuous stirring (500 rpm). After 1 h, the samples were centrifuged (14 000 rpm, 15 min) and the filtered (0.22 μm, PTFE) supernatant was analysed for its oligosaccharide, monosaccharide and dehydration product concentration.

### Synthesis of amorphous cellulose

Amorphous cellulose was synthesized through precipitation of Avicel PH-101 dissolved in a 50/50 mixture NaSCN (Sigma-Aldrich, ≥98%) and ethylenediamine (Sigma-Aldrich, ≥99.5%) in accordance with a procedure described elsewhere.^51^

### Adsorption of cellobiose

Adsorption of cellobiose (20 mM) onto C-350-SO_3_H-HT (100 mg catalyst per 1 mL solution) was performed in 2.5 mL centrifuge vials placed in an Eppendorf Thermomix Comfort set to 25 °C and 4000 rpm. After a set amount of time, the vials were centrifuged (14 000 rpm, 15 min) and the filtered (0.22 μm, PTFE) supernatant was analysed for its cellobiose concentration.

### Recyclability experiments

During recyclability experiments the catalyst was recovered from the mixture containing unreacted cellulose, by dissolving the latter during 80 min in 72% H_2_SO_4_ (15 mL per 1 g of powder mixture) heated to 30 °C. Subsequently, the catalyst was filtered from the solution and washed with 1 L of deionized water at room temperature and 2 L of hot (90–95 °C) deionized water. To ensure complete removal of cellulose the dissolution procedure was performed twice.

### Compositional analysis

Compositional analysis was performed in accordance with the methodologies described by NREL.^48,52–54^ The mass balance for the compositional analysis of the spruce fir wood was closed to 106%. The spruce fir wood was chopped on the 15^th^ of September 2015 in the forest of Würenlingen, Switzerland.

## Results and discussion

### Sulfonated carbon catalyst

The sulfonated carbon catalyst utilized in this study was prepared by carbonization of cellulose at 350 °C (denoted C-350) and sulfonation of the resulting carbonaceous material (denoted C-350-SO_3_H) using oleum (20% SO_3_/H_2_SO_4_). Sulfonation of C-350 led to the introduction of both hydrothermally stable and instable sulfonic acid groups (–SO_3_H: 2068 ± 57 μmol g^−1^). Hydrothermal pre-treatment (180 °C, 16.5 h) of the sulfonated materials removed the instable groups and resulted in a material (denoted C-350-SO_3_H-HT) with 869 ± 29 μmol g^−1^ stable –SO_3_H groups. The chemical identity of sulphur species in the catalyst was validated with Fourier-transform infrared (FTIR) spectroscopy. The FTIR spectra (Fig. S1A†) show two clearly identifiable absorption bands at 1040 and 1168 cm^−1^, assigned to the symmetric and asymmetric stretching modes of O=S=O in –SO_3_H groups, respectively.^55^ To verify that the carbon framework of C-350-SO_3_H was hydrothermally stable its crystalline and molecular carbon structure were characterized with X-ray diffraction (XRD) and Raman spectroscopy, respectively. Both the XRD patterns (Fig. S1.†B) and the Raman spectra (Fig. S1.†C) confirmed that the amorphous carbon structure did not undergo structural transformation during hydrothermal treatment. Measurement of the specific surface area (SSA) using Krypton as an adsorbate (Fig. S1D†) further showed that the sample preserved its textural properties (Table S1†) during the treatment in hot-compressed water.

### Semi-batch reactor

**Fig. 1:**
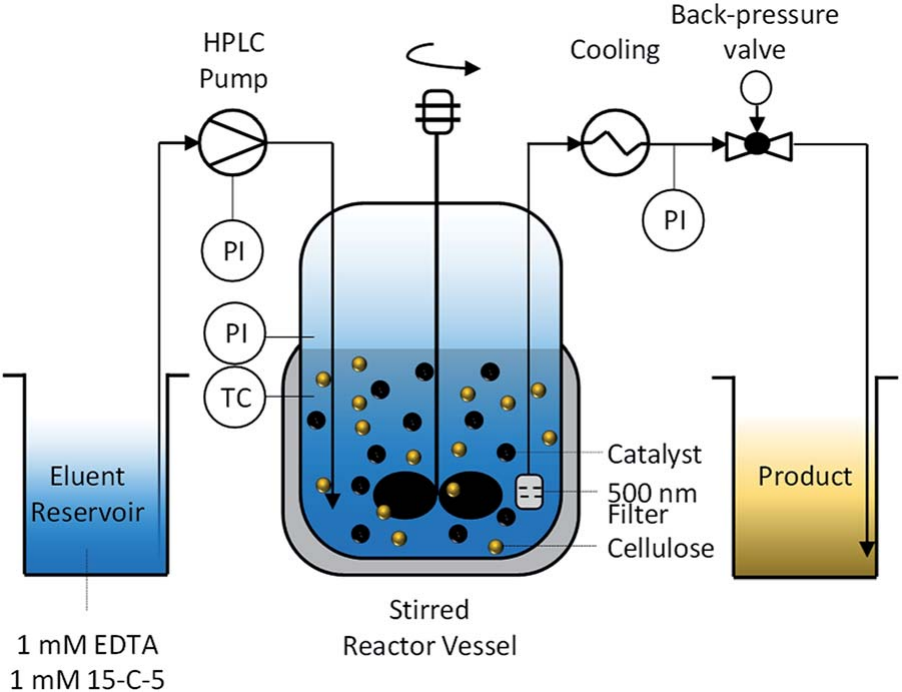
Semi-batch process for hydrolysis of cellulose and softwood using sulfonated carbon catalysts.

To enable the hydrolysis of cellulose under continuous-flow conditions and using solid acid catalysts, we developed a semi-batch process (Fig. 1).

The reactor is composed of a continuously stirred tank, connected at the inlet to an HPLC pump and at the outlet to a back-pressure valve. To retain the unreacted substrate and the catalyst, the reactor effluent was filtered through a 500 nm porous metal-frit installed at the outlet of the reactor. To prevent the frit from clogging with particles, it was installed close (2 mm) to the impeller. We found that this design feature was crucial for maintaining a filter surface free of deposited particles and thus for maintaining a low pressure drop over the frit. We attribute this effect to the shear forces at the surface of the filter originating from the turbulent fluid environment induced by the rotating impeller. The formation of a turbulent fluid environment is confirmed by considering the impeller Reynolds number (*N*_Re_(600 rpm) = 2.5 × 10^6^) for the system which is two orders of magnitude greater than the boundary value^56^ for fully turbulent fluids in stirred tank reactors.

The continuous product removal is an essential feature of the reactor system that enables decoupling the residence times of the substrate and the hydrolysis products (mean residence time, *τ* = 35 min). Thereby the undesired decomposition reactions^57^ of monosaccharides that typically limit the attainable reaction yields can be minimized.

Due to the tendency of the sulfonated carbon catalyst to deactivate in presence of cationic impurities,^39^ the eluent was enriched with 1 mM 15-crown-5 polyether (15-C-5) and ethylenediaminetetraacetic acid (EDTA) as complexation agents for the mono- and divalent cations, respectively. The cellulose used in this study had an ash content of 0.2% and is therefore expected to liberate cations into the reaction medium during the course of the reaction.

### Hydrolysis pathways

**Scheme 1:**
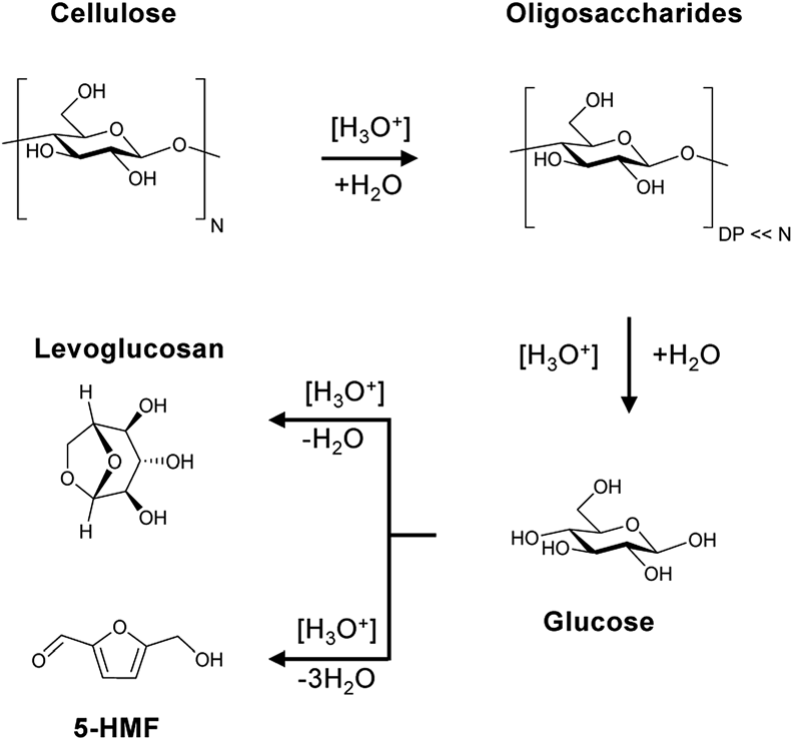
Reaction pathway for cellulose, oligosaccharides and monosaccharides in the presence of Brønsted acids.

The Brønsted acid catalysed hydrolysis of cellulose is initialized with the formation of soluble and insoluble oligosaccharides (Scheme 1). It is well established in literature that solubility is defined by the degree of polymerization (DP).^58^ Whereas oligosaccharides with a DP of 2–6 are soluble in water at room temperature, glucans with 7–13 monomeric units are only partially soluble in hot compressed water. The formed oligosaccharides can subsequently undergo secondary hydrolysis to form glucose. The formation of aldose (mannose and galactose) and ketose (fructose) isomerization products of glucose is attributed to a Lobry de Bruyn-van Ekenstein transformation.^59^ Finally, the formed monosaccharides can undergo intra- or inter-molecular dehydration to form levoglucosan and 5-(hydroxymethyl)furfural (5-HMF), respectively. This typically unwanted side reaction of C_6_ monosaccharides should however be limited by the continuous product removal from the semi-batch reactor.

Cellulose and the sulfonated carbon catalysts are both insoluble in water at room temperature. The question thus arises how the reaction between the solid acid and cellulose should proceed. It has been proposed that the reaction occurs through a collision induced event^29,30,41^ or through adsorption of the substrate onto the catalysts surface.^35,36^

It is however important to highlight that the formation of soluble reaction products could also occur *via* homogeneously catalysed reactions. For example, it is well known that cellulose readily undergoes auto-hydrolysis,^8,60^ albeit, typically at higher temperatures than used in this study. Hydrolysis could also be homogeneously catalysed by protons provided by the dissociation of EDTA. Our initial experiments therefore aimed at elucidating the contribution of homogeneous reaction pathways to the hydrolysis of cellulose.

**Fig. 2:**
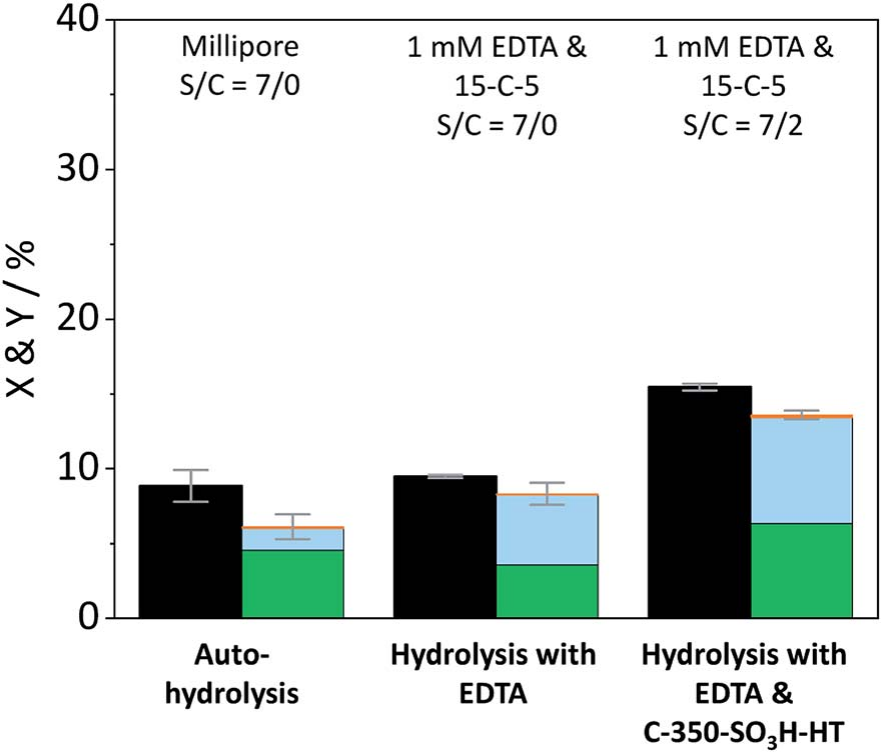
Conversion (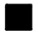) and yields of oligosaccharides (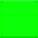), monosaccharides (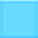) and dehydration products (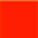) achieved with pristine cellulose in presence and in absence of EDTA and C-350-SO_3_H-HT. Reaction conditions: 165 °C, 6.5 h, 3 wt% cellulose, *τ* = 35 min, impeller speed = 600 rpm.

The contribution of auto-hydrolysis was determined through an experiment in which the reactor was only charged with cellulose and Millipore water was used as the solvent. Even at the mild temperature (165 °C) employed in this work, auto-hydrolysis led to 8.9% conversion (*X*) to yield (*Y*) 4.6% oligosaccharides, 1.5% monosaccharides and 0.1% dehydration products (Fig. 2).

When the eluent was enriched with 1 mM EDTA and 15-C-5, the conversion of cellulose increased marginally to 9.5% due to the higher concentration of [H_3_O^+^] ions present in this case (pH 3). Furthermore, the formed oligosaccharides increasingly underwent secondary hydrolysis. Oligosaccharides, monosaccharides, and dehydration products were formed with 3.6%, 4.7%, and 0.1% yield. The slightly increased conversion confirms that the protons liberated by EDTA (p*K*_a_ = 0, 1.5, 2.0, and 2.66)^61^ contributed to the formation of soluble reaction products. The overall increase was however limited due to the fact that the hydrolysis reaction is initiated by the protonation of the weakly basic glycosidic oxygen^15^ and thus, typically, requires the use of high concentrations of strong Brønsted acids (p*K*_a_ ≤ −3),^37^ to achieve acceptable reaction rates.

In presence of both EDTA and the sulfonated carbon catalyst, the conversion of cellulose increased further to 15.5%. Over the course of the reaction, oligosaccharides, monosaccharides and dehydration products were formed with 6.3%, 7.1%, and 0.2% yield (Fig. 2). Notably, even in presence of the sulfonated carbon catalyst the yield of dehydration product remained low. This confirms that the continuous product removal from the semi-batch reactor effectively suppressed secondary reactions of the formed monosaccharides.

The fact that the conversion increased when the hydrolysis was performed in presence of the sulfonated carbon catalyst, suggests that cellulose can additionally undergo a heterogeneously catalysed reaction pathway. However, despite the presence of strong Brønsted acid sites in the catalyst, the conversion and achieved yields were low as compared to those reported in previous studies (50.4–74.5%).^18,35,42^ We attribute this finding to the fact that the reaction with a solid acid catalyst is strongly inhibited by the substrates insolubility and therefore predominantly occurs under participation of leached active sites from the catalyst. The sulfonated carbon catalyst employed in this work leached only 16% of its sulfonic acid groups during the reaction, which corresponds to a hypothetical H_2_SO_4_ concentration of 1 mM in the reaction medium. The release of H_2_SO_4_ into the reaction medium would invoke a further increase in the concentration of protons (pH 2.7), leading to an acceleration of homogeneously catalysed reactions. Thus, the true contribution of a heterogeneously catalysed reaction to the conversion of the substrate could not be determined accurately. However these experiments clearly show that homogeneous reaction pathways (auto-hydrolysis, hydrolysis by EDTA and hydrolysis by leached species) significantly contribute to the conversion of cellulose.

Following from the hypothesis that the reaction between cellulose and the catalyst occurs *via* a collision-induced event,^29,30,41^ we investigated the effect of the collision frequency on the hydrolysis reaction rate. We anticipated that the collision frequency between the substrate and the catalyst should be correlated to the degree of turbulence in the reaction medium. To test this hypothesis we performed several experiments in which we varied the impeller rotational speed (75–600 rpm). Surprisingly, the conversion and the total yield remained constant irregardless of the impeller speed (Fig. S3†). This consequently suggests that the initial formation of soluble oligosaccharides *via* a heterogeneously catalysed reaction pathway does not proceed *via* a collision-induced event, or that the collision of the substrate with the catalyst is not the rate determining step in the reaction mechanism.

### The effect of ball-milling on the crystal structure of cellulose

The comparatively low yields achieved with pristine cellulose are a direct manifestation of its pronounced chemical recalcitrance. The recalcitrance is linked to the presence of an extended intra- and inter-chain hydrogen bonding network that facilitates the formation of partially crystalline domains.^3^ Glycosidic bonds in crystalline domains are shielded from the chemical environment and are thus significantly less reactive^62^ than such in para-crystalline domains. The latter are commonly referred to as amorphous domains and the two terms are used interchangeably in this study.

The higher reactivity of glycosidic bonds in para-crystalline domains suggests that amorphization of cellulose should be an effective strategy to enhance its propensity to undergo hydrolysis. Among the different pre-treatments used for enhancing the reactivity of cellulose, ball-milling has been identified as one of the most effective.^63^ Ball-milling is known to affect various structural properties of cellulose, including the particle size and crystallinity.^64^ To understand how structural properties evolved with treatment time and how these correlated with reaction yields, we prepared samples subjected to different durations of ball-milling (denoted BM-x h).

**Fig. 3:**
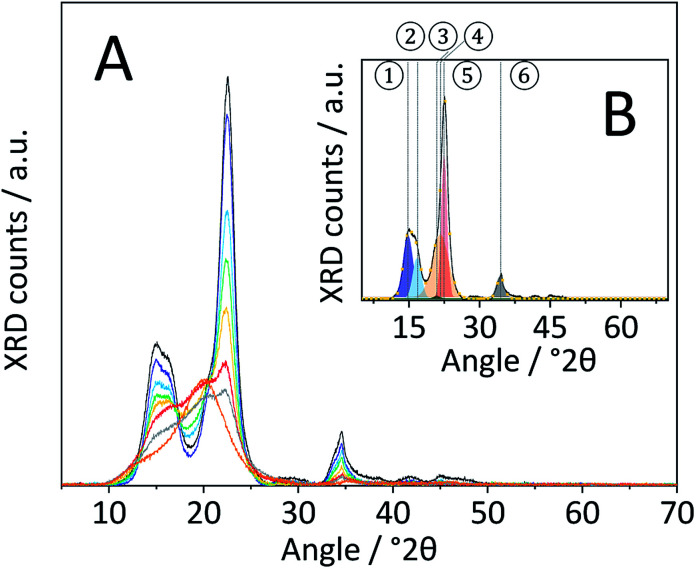
(A) Powder X-ray diffraction patterns of pristine (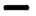), BM-1 h (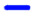),BM-4 h (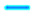), BM-8 h (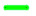),BM-18 h (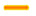),BM-54 h (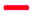),BM-18 h + 5% H_2_O (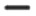) and amorphous cellulose (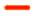). (B) Powder X-ray diffraction pattern of pristine cellulose (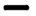), the fitted ① – (1 0 1) (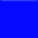), ② – (1 0 1̄) (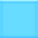), ③ – (0 2 1) (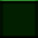), ④ – para-crystalline (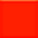), ⑤ – (0 0 2) (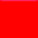), ⑥ – (0 0 4) (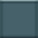) peaks and sum of the fitted peaks (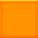).

Changes in the crystal structure were assessed by measuring the XRD patterns of the various samples. The diffraction pattern of pristine cellulose (Fig. 3A) shows three peaks centred at 2*θ* = 15, 22.5 and 34.5° that are characteristic of the naturally occurring cellulose allomorph. The three peaks form due to overlapping diffractions associated with the (1 0 1), (1 0 1̄), (0 2 1), (0 0 2) and (0 0 4) crystal planes.^49^ Additionally, it is generally recognized that a broad peak at 2*θ* = 20° is characteristic of para-crystalline domains.^49^ This assignment was confirmed by measuring the diffraction pattern of an amorphous cellulose sample (Fig. 3A).

The amorphization during ball-milling can be directly inferred from the decreasing intensity of the peaks at 2*θ* = 15, 22.5 and 34.5° and the increase in intensity at 2*θ* ≈ 20°. To quantify the change in crystallinity, the diffraction patterns were deconvoluted into the contributions from the different crystal planes (Fig. 3B). A relative measure of crystallinity can then be calculated as the quotient of the total area of crystalline reflexes to the total area of the diffraction pattern.^49^ Following this methodology the crystallinity index (CI) of pristine cellulose was determined to be 65.3%, which is consistent with values reported previously.^49^ The CI of pretreated cellulose decreased monotonously with the duration of ball-milling (Fig. 5) and reached 56.5% in the sample BM-54 h. The CI initially rapidly decreased and then began to plateau at longer treatment times. This is tentatively attributed to the tendency of the substrate to adhere to the milling media at longer treatment times. The substrate layer is expected to dampen the mechanical forces during impact, which would consequently reduce the efficiency of the pre-treatment. Similar observations have also been made by Rinaldi and co-workers.^65^

Nonetheless, the determined CI's confirm that ball-milling promoted the amorphization and that this structural transformation was most pronounced in samples subjected to longer treatment times. The increase in structural heterogeneity during ball-milling is also exemplified by considering the full width at half maximum (FWHM) of the deconvoluted peaks. It is commonly accepted in literature that peak broadening in the diffraction pattern of cellulose is related to an increased amorphous content.^49^ With the exception of the (0 0 2) diffraction, the FWHM of the deconvoluted peaks indeed increased with prolonged ball-milling duration (Table S2†). Even in samples subjected to prolonged pre-treatment, but displaying only minor changes in the CI (BM-18 h and BM-54 h), ball-milling lead to continued peak broadening, that is to say amorphization.

It should be noted that peak broadening may also be related to changes in the sizes of crystallites. To assess this effect on the FWHM, the crystallite size in the various samples was estimated with the Scherrer equation. The average crystallite size determined from the crystal planes in pristine cellulose was 4.8 nm, which is consistent with values reported in literature.^49^ The crystallite size in ball-milled samples (Table S3†) initially decreased with the duration of ball-milling and remained constant at 3.5 nm at longer treatment times (18 h and 54 h). Consequently, it can be concluded that at least a part of the increased FWHM is associated with a reduction in the crystallite size. However, it is important to point out that ball-milling induced dislocations, faults or stress in the lattice would likewise contribute to peak-broadening.

Besides changing the crystallite size, ball-milling also had a pronounced effect on the particle size distribution. After 1 h of ball-milling, the mean of the particle size distribution (*d*_50,V_) changed from 50.8 μm to 22.4 μm. Interestingly, longer treatment times did not lead to a further reduction in the particle size distribution (Fig. S4C†), but to the formation of increasingly spherical particles (Fig. S4B†).

Although ball-milling promoted amorphization, the CI showed that the samples retained a substantial fraction of their crystallinity. Therefore in a next step, means to enhance the amorphization during ball-milling were investigated. Based on the ability of water molecules to form H-bonds with cellulose,^66^ it was postulated that the addition of water during ball-milling may aid in disrupting crystalline structures. To test this hypothesis, cellulose was ball-milled for 18 h with prior addition of 2–25 wt% H_2_O. As postulated, the CI index decreased when up to 10 wt% H_2_O was added during ball-milling (Fig. S5A†). The lowest CI index (52.0%) was achieved when 5% H_2_O was added. At higher water loadings the CI index increased and at 25 wt% approached the value for pristine cellulose. The amount of added water also had a pronounced effect on the particle size (Fig. S5C†) and morphology (Fig. S5B†). The addition of 2–10% H_2_O promoted the formation of larger and highly spherical particles, whereas at 25 wt% H_2_O added the characteristics of the particle size distribution approached the values found for pristine cellulose. The latter effect is related to the formation of a viscous paste that retarded the motion of the grinding media.

### The effect of ball-milling on the hydrogen bond network

**Scheme 2:**
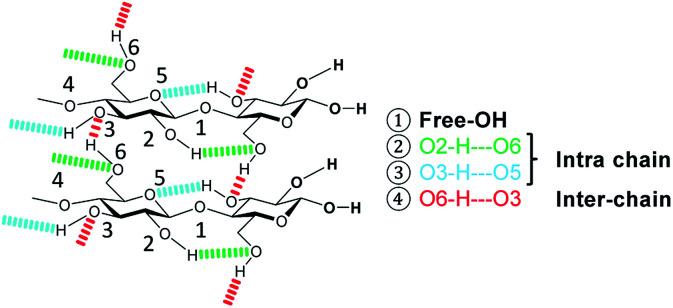
Hydrogen bonding in cellulose.

The formation of crystalline domains in cellulose is facilitated by an extended inter- and intra-chain hydrogen bonding network. Therefore, changes in the crystallinity should be accompanied by alterations in the hydrogen bond network (Scheme 2).

**Fig. 4:**
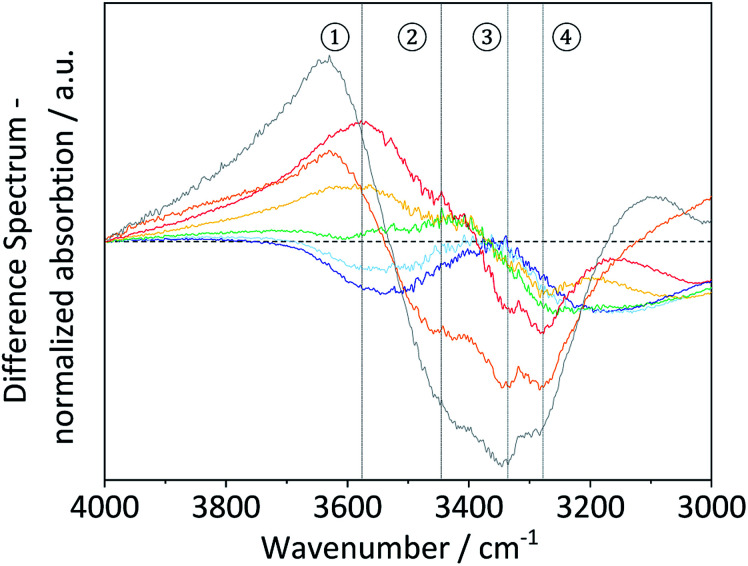
FTIR difference spectra of BM-1 h (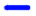), BM-4 h (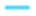), BM-8 h (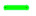), BM-18 h (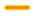), BM-54 h (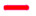), BM-18 h + 5% H_2_O (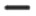) and of amorphous cellulose (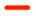).

To study these changes, FTIR spectra of the different samples in the 4000–3000 cm^−1^ wavenumber region (Fig. S7†) were recorded. According to literature, absorption in this region can be assigned to the *ν*(O–H) stretching vibration of inter- (O6–H–O3 – 3310–3230 cm^−1^) and intra-chain (O3–H–O5 – 3375–3340 cm^−1^ and O2–H–O6 – 3460–3405 cm^−1^) hydrogen bonded as well as free –OH groups (3580–3550 cm^−1^).^67^ Visualization of changes in the spectra was accomplished by determining the normalized difference spectra of the pretreated samples, relative to pristine cellulose (Fig. 4). The difference spectra of the samples BM-18 h and BM-54 h showed two strongly overlapping negative peaks centred at 3345 and 3283 cm^−1^, assigned to the valence vibration of intra-chain O3–H–O5 and inter-chain O6–H–O3 H-bonded –OH groups, respectively. The fact that the difference spectra had negative intensity indicates that the O3–H–O5 and O6–H–O3 H-bonds had been partially disrupted in these samples. Additionally, a broad positive peak centred at 3576 cm^−1^ with a shoulder of weak intensity at 3445 cm^−1^ was formed in these samples. The peaks are assigned to the stretching vibration of free and of intra-chain (O2–H–O6) bonded –OH groups, respectively. The positive nature of the peak at 3576 cm^−1^ shows that these samples contained more free –OH groups, as would be expected from the disruption of O3–H–O5 and inter-chain O6–H–O3 H-bonded –OH groups.

Similar structural changes in the H-bond network were also found in the amorphous cellulose and the sample that was ball-milled with 5% H_2_O. However, in both samples the negative absorption peaks assosciated with O3–H–O5 and O6–H–O3 H-bonded –OH groups had higher intensity, suggesting that these H-bonds had been disrupted to a greater extent. These results thus demonstrate that besides reducing the crystallinity and the particle size, ball-milling has a measurable effect on the nature of the H-bond network. Furthermore, this effect is most pronounced in samples that experienced the highest reduction in crystallinity, which confirms that amorphization occurs hand in hand with a disruption of the H-bond network.

### The interrelation between structure and specific reactivity

**Fig. 5:**
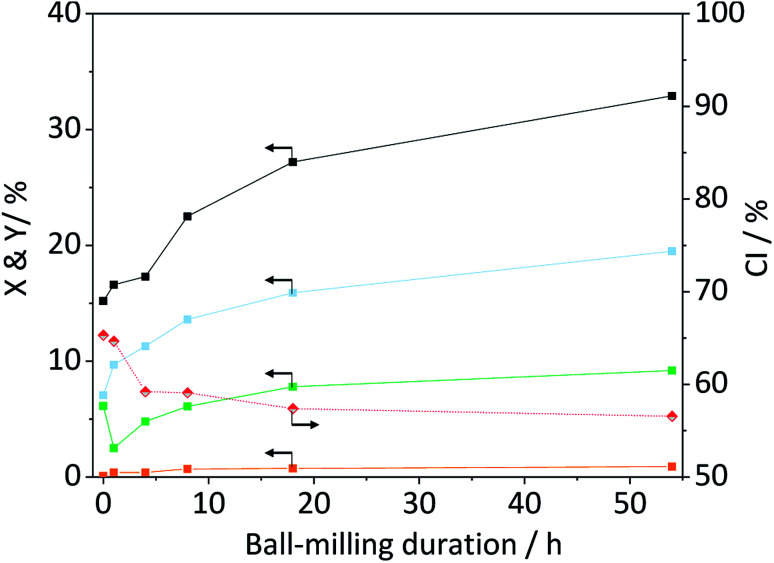
Crystallinity index (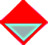), conversion (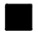) and yields of oligosaccharides (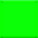), monosaccharides (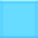) and dehydration products (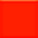) achieved with ball-milled cellulose in presence of C-350-SO_3_H-HT. Reaction conditions: 165 °C, 6.5 h, 3 wt% BM-cellulose, S/C = 7/2, 1 mM EDTA & 15-C-5, *t* = 35 min, impeller speed = 600 rpm.

With the aim to identify structure–reactivity relations, we progressed to studying the hydrolysis of the pretreated cellulose samples. Fig. 5 shows the conversion and yields achieved with ball-milled cellulose in presence of C-350-SO_3_H-HT. The conversion initially increased with the duration of ball-milling and then plateaued at longer treatment times. Interestingly, the CI's of the samples displayed a similar trend. That is to say, the CI initially decreased and then reached a constant value at longer treatment times.

The trends in the reaction yields and in the CI's, suggests that the degree of crystallinity is a descriptor for the specific reactivity of cellulose. This hypothesis is also supported by the results obtained with wet ball-milled samples. In these samples, it was found that an increase/decrease in the CI (Fig. S5A†) was directly correlated with a decrease/increase in the total reaction yields (Fig. S6†). The sample ball-milled with 5% H_2_O had the lowest CI (52.0%) and achieved the highest level of conversion (34.0%). To substantiate the hypothesis that reactivity is related to the CI, we tested the propensity of the amorphous cellulose (CI = 20.1%) to undergo hydrolysis. To our surprise the achieved conversion and yields (Table S3†) were comparable to the ones achieved with cellulose that was ball-milled for 18 h (CI = 57.4%). This clearly indicates that other factors must be influencing the reactivity of cellulose. Besides the crystallinity, the reactivity may also be strongly determined by the particle size. Smaller particles would be expected to show higher reactivity because the surface area where a reaction could occur would be larger. This effect may be responsible for the lower reactivity of the amorphous cellulose. Although the sample featured the lowest CI of all tested samples, the mean of its particle size distribution (*d*_50,V_) was nearly four times as large as that of the BM-18 h (Table S3†).

To further elucidate the structural origin of reactivity, we investigated the solubility of the pretreated samples. It is well known that ball-milling can induce mechanochemistry *via* bond breakage and formation.^68^ On that basis, we hypothesized that the enhanced reactivity of ball-milled cellulose could be related to the cleavage of glycosidic bonds, leading to the formation of smaller and therefore partially soluble oligosaccharides. The solubility of pristine cellulose in Millipore water at 25 °C was 0.05%, and thus, negligible. With increasing ball-milling duration, the solubility monotonously increased and reached 0.79% after 54 h (Table S3†). The soluble fraction was composed of oligosaccharides (94%) and monosaccharides (6%). No traces of dehydration products (5-HMF and levoglucosan) could be detected in any of the samples. Analysis of the soluble fraction using liquid chromatography coupled to a mass spectrometer (LC-MS) enabled identification of oligosaccharides with a DP of up to 9 (Fig. S8B†). We do not exclude that larger oligosaccharides formed during ball-milling. However, due to the dependence of solubility on the DP it is improbable that larger oligosaccharides dissolved in sufficiently high concentration to enable reliable analysis. We stress that we found oligosaccharides of comparable size (DP = 8) in the soluble fraction of pristine cellulose (Fig. S8A†), although at lower concentration.

**Fig. 6:**
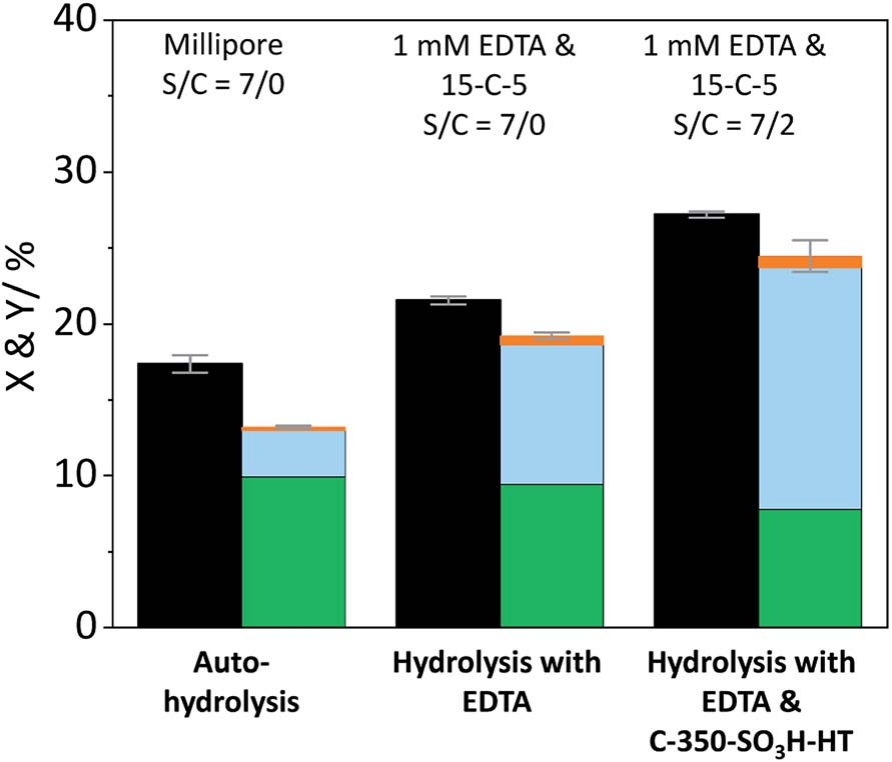
Conversion (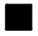) and yields of oligosaccharides (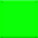), monosaccharides (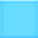) and dehydration products (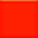) achieved with 18 h ball-milled cellulose in presence and in absence of EDTA and C-350-SO_3_H-HT. Reaction conditions: 165 °C, 6.5 h, 3 wt% BM-18 h, *τ* = 35 min, impeller speed = 600 rpm.

The formation of soluble oligosaccharides confirms that ball-milling indeed induced glycosidic bond cleavage. The increased solubility undoubtedly aids in the conversion of cellulose during the subsequent hydrolysis in the semi-batch reactor. Additionally, it is reasonable to assume that the solubility of the pretreated samples was even higher under the conditions in the semi-batch reactor (165 °C). However, due to the tendency of cellulose to undergo auto-hydrolysis at elevated temperatures it is not possible to measure the solubility of samples under the experimental conditions. Nevertheless, we investigated if the enhanced reactivity can be ascribed to an enhanced tendency of cellulose to undergo homogeneous hydrolysis pathways. Fig. 6 shows the conversion and yields achieved with the sample BM-18 h in presence and in absence of EDTA and the sulfonated carbon catalyst. In comparison with pristine cellulose (Fig. 2) the conversion through auto-hydrolysis increased from 8.9% to 17.4%. The major reaction products were the same as with pristine cellulose; oligosaccharides (*Y* = 9.9%) that only partially underwent secondary hydrolysis to monosaccharides (*Y* = 3.1%). When the hydrolysis was performed in presence of EDTA the conversion (*X* = 21.6%) further increased and the formed oligosaccharides (*Y* = 9.4%) increasingly underwent secondary hydrolysis to monosaccharides (*Y* = 9.2%). In presence of both EDTA and the sulfonated carbon catalyst, the conversion reached 27.2% (15.7% with pristine cellulose). Thus, as during experiments with pristine cellulose the conversion increased by approximately 6% when the reaction was performed in presence of the sulfonated carbon catalyst.

The pathway experiments clearly exemplify that the higher specific reactivity of ball-milled cellulose can be explained with an enhanced tendency of the substrate to undergo homogeneous hydrolysis (auto-hydrolysis and hydrolysis by EDTA). The enhanced reactivity can be rationalized with the increased amorphous content (lower CI) and the higher propensity of the amorphous fraction to undergo homogeneous hydrolysis. The preferential hydrolysis of glycosidic bonds in amorphous domains by homogeneous reactions is confirmed by the CI determined for the unconverted residues. The CI increased from 57.4% to 72.1% in the unconverted residue recovered after the auto-hydrolysis experiment with BM-18 h (Fig. S9B†). An increase in the CI was also found in the unconverted residue recovered after experiments with pristine cellulose (Fig. S9A†). These results thus conclusively explain why ball-milled cellulose is often found to exhibit a higher specific reactivity during a solid acid catalysed hydrolysis. The higher reactivity does not result from an enhanced reaction with the solid acid, but rather from the higher tendency of the substrate to undergo homogeneously catalysed reactions. Thus, homogeneous reaction pathways play a critical role in the hydrolysis of cellulose over solid acid catalyst. It is important to point out that in many studies dealing with the solid acid catalysed conversion of cellulose, the contribution of homogeneously catalysed reaction is overlooked. The findings presented here, however show that even under the comparatively mild reaction conditions chosen in this study, amorphized cellulose readily undergoes homogeneously catalysed hydrolysis (auto-hydrolysis and hydrolysis by EDTA).

### Mechanocatalytic depolymerization of cellulose

Despite the higher yields achieved with ball-milled cellulose, the majority of product formation occurs *via* homogeneously catalysed reactions and only to a limited extent *via* reaction with the sulfonated carbon catalyst.

**Fig. 7:**
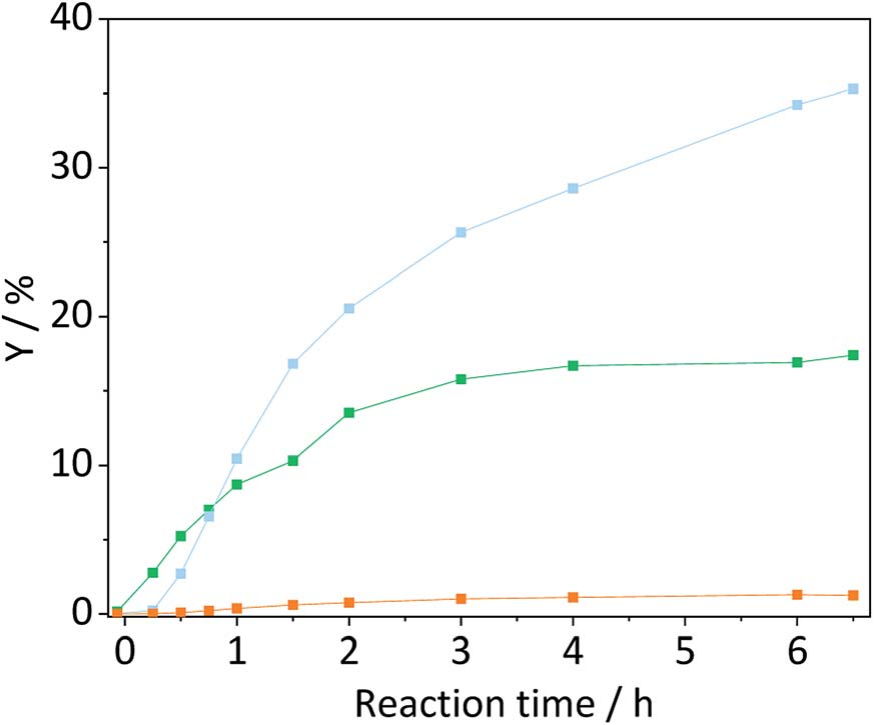
Yields of oligosaccharides (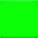), monosaccharides (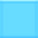) and dehydration products (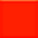) achieved with CBM-7/2 treated cellulose. Reaction conditions: 165 °C, 6.5 h, 3 wt% CBM-7/2, 1 mM EDTA & 15-C-5, *τ* = 35 min, impeller speed = 600 rpm.

A truly heterogeneously catalysed hydrolysis should however occur during the concerted ball-milling (CBM) of cellulose with a solid acid. To investigate the efficacy of the sulfonated carbon catalyst in facilitating a mechanocatalytic depolymerization and to understand the influence on the structure and reactivity of cellulose, we subjected the two powders to CBM treatment (S/C = 7/2, 300 rpm, 18 h). Fig. 7 shows the yields in oligosaccharides, monosaccharides and dehydration products achieved with CBM-7/2 treated cellulose. Compared with cellulose subjected to individual ball-milling, the CBM treated cellulose much more readily underwent conversion to yield 17.4%, 35.3% and 1.2% oligo-, monosaccharides and dehydration products during hydrolysis in the semi-batch reactor.

To elucidate the phenomena leading to the enhanced reactivity, structural changes in the CBM treated cellulose were characterized. In a first step the XRD patterns of the CBM treated samples were recorded and the respective contribution of cellulose to these was determined. The latter was accomplished by assuming that the diffraction pattern of the powder mixture can be described as a weight-fraction weighted linear combination of the diffraction patterns of the individual components.^69^ This approach was verified by comparing the measured and the calculated diffraction pattern of a 7/2 physical mixture of individually ball-milled cellulose and individually ball-milled C-350-SO_3_H-HT (Fig. S10†).

**Fig. 8:**
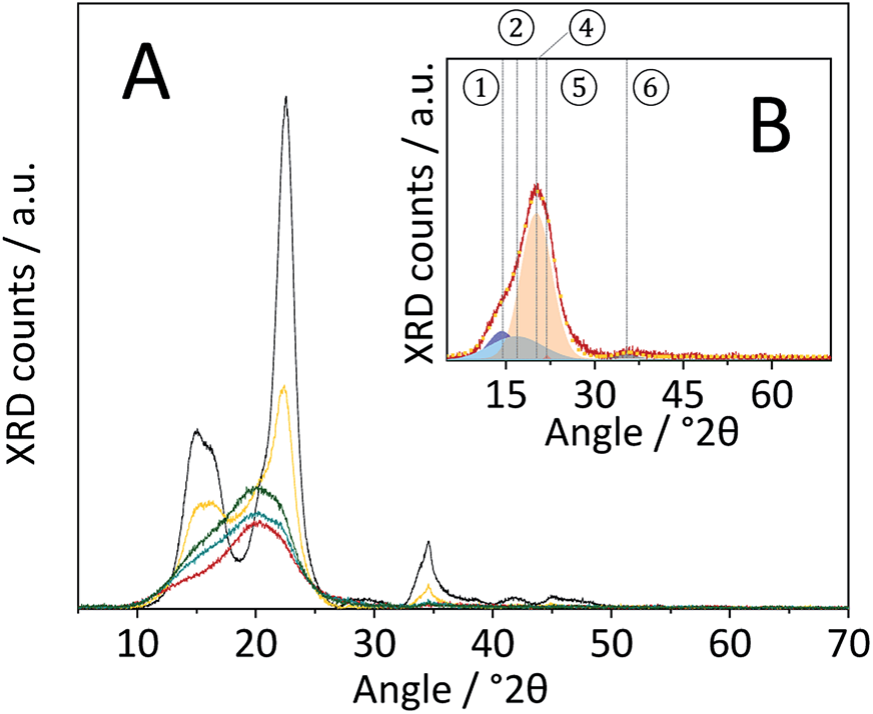
(A) Powder X-ray diffraction patterns of pristine (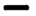), BM-18 h (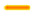), CBM-7/2 (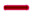), CBM-7/2-no sulfonation (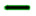) and CBM-7/2-deactivated catalyst (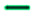). (B) Powder X-ray diffraction pattern of CBM-7/2 (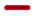), the fitted ① – (1 0 1) (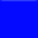), ② – (1 0 1̄) (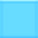), ④ – para-crystalline (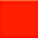), ⑤ – (0 0 2) (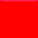), ⑥ – (0 0 4) (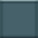) peaks and sum of the fitted peaks (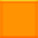).

The diffraction pattern of CBM treated cellulose (Fig. 8A) was composed of a single broad peak centred at 2*θ* ≈ 20°, previously assigned to para-crystalline structures. The broad peak featured a shoulder at 2*θ* ≈ 15° associated with overlapping diffractions from the (1 0 1) and (1 0 1̄) crystal planes. A very weak signal detected at 2*θ* = 34.5° is associated with the (0 0 4) diffraction. Comparing the diffraction patterns of CBM-treated and individually ball-milled cellulose, illustrates that the former experienced a much higher degree of amorphization. Correspondingly, the CI determined from the deconvoluted peak areas of CBM-treated cellulose (31.6%) was significantly lower than of individually ball-milled cellulose (57.4%).

### The role of catalyst surface functionalities during mechanocatalytic depolymerization

To understand the processes involved in amorphizing cellulose and the role of the catalyst's surface functionalities in these, the CBM treatment was repeated with carbon materials featuring variations in their surface chemistries. The CBM treatment was repeated with firstly a non-sulfonated carbon material (denoted CBM-7/2-no sulf.) and secondly with a deactivated sulfonated carbon catalyst (denoted CBM-7/2-deac.). The deactivated catalyst was prepared by exchanging the protons of the sulfonic acid groups with cations, *via* three consecutive ion-exchange treatments with an aqueous solution containing commonly occurring cationic impurities (Na^+^, K^+^, Ca^2+^ and Mg^2+^ 0.25 wt% each). Interestingly, in both cases the CBM treatment led to comparable degrees of amorphization as when the as synthesized C-350-SO_3_H-HT was used in the treatment (Fig. 8A). The corresponding CI's of CBM-treated cellulose with the non-sulfonated or the deactivated materials were 35.9% and 34.7%, respectively. The fact that similar reductions in crystallinity occurred regardless of the material's surface chemistry, indicates that amorphization is not solely induced by the action of the sulfonic acid groups, *i.e. via* glycosidic bond cleavage, but may rather be related to an interaction between the carbon material and cellulose. Furthermore, because deep amorphization could not be achieved by physically mixing the two solids (Fig. S10†), the interaction between the two powders appears to be facilitated by the CBM treatment.

Previous studies have provided compelling evidence for the ability of poly-aromatic systems in carbon materials to adsorb oligosaccharides from aqueous solutions.^70,71^ The adsorption process occurs on hydrophobic sites on the carbon material (polyaromatic domains) and is driven by entropically favoured hydrophobic interactions combined with CH–π hydrogen bonding.^72^ The affinity of oligosaccharides for carbons has been claimed to be higher than the affinity between oligosaccharides.^72^ Consistent with these studies, adsorption tests performed in the scope of this study showed that the sample C-350-SO_3_H-HT had an adsorption capacity of 0.79 mg cellobiose g_cat_^−1^ (Fig. S11†). It may therefore be possible that the CBM treatment led to sufficient proximity between the carbon material and the sulfonated carbon catalyst to facilitate adsorption and thereby amorphization. In an effort to validate this analysis, a FTIR spectroscopy based investigation of the liquid-phase and mechanochemical adsorption process was performed. Spectroscopic evidence of the adsorption process by comparing the position of the *ν*(C–H) absorption band in free and adsorbed cellobiose/cellulose could however not be established due to the to the low adsorbate loading and the strong IR absorptivity of the carbon material.

**Fig. 9:**
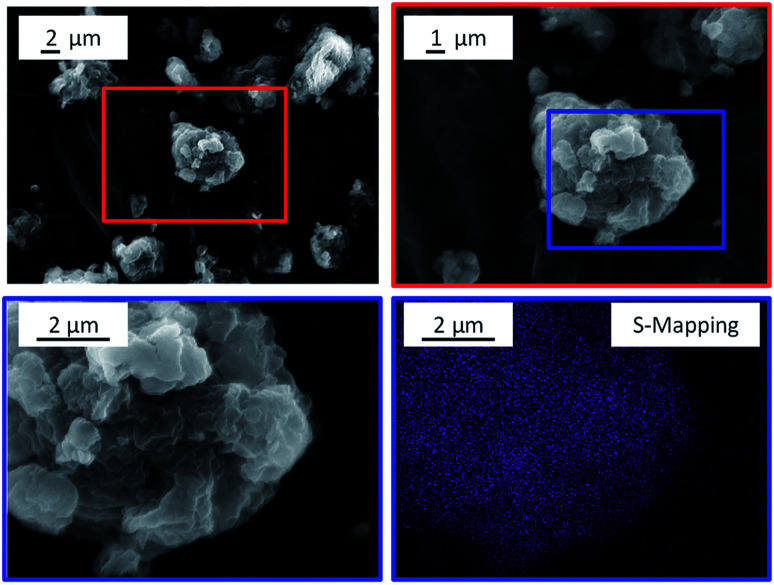
SEM images and sulphur EDX mapping of CBM-7/2.

The pronounced affiliation between the substrate and the catalyst can however be seen in the SEM images of the CBM-7/2 mixture (Fig. 9). The images show strongly aggregated particles in which the grain boundaries of the substrate and the catalyst cannot be identified. To visualize the strongly aggregated nature of the powder mixture, energy-dispersive X-ray spectroscopy (EDX) was employed to map the distribution of sulphur (originating solely from the catalyst) within the particles. The EDX mapping shows that sulphur is evenly distributed within the particles, which indicates that the CBM treatment promoted the formation of a solid–solid suspension of the substrate and the catalyst. The strongly affiliated nature of the CBM components is also supported by the fact that the particle size distribution of the mixture was unimodal (Fig. S12†) and had a mean particle size (13.5 μm) that was nearly half as large as that of individually ball-milled cellulose (24.6 μm). The higher degree of amorphization as well as the further reduction in particle size achieved during CBM, as compared to standard ball-milling, is thus tentatively attributed to an interaction of the two powders facilitated by the strong mechanical forces exerted upon them during CBM treatment.

Although similar degrees of amorphization were achieved during the CBM treatment with C-350, C-350-SO_3_H-HT and C-350-SO_3_H-HT-deac, the samples displayed fundamentally different reactivity (Table S4†). When the non-sulfonated carbon was used for the CBM treatment, the obtained yields in oligo-, monosaccharides and dehydration products were 12.8%, 12.3% and 0.3%, at 26.9% conversion, respectively. The conversion and yields are comparable to those obtained when hydrolysing individually ball-milled cellulose in absence of the sulfonated carbon catalyst (Fig. 6). Thus, in this case, conversion occurred predominantly *via* homogeneously catalysed pathways (auto-hydrolysis and hydrolysis by EDTA). Similarly, the conversion and yields increased only marginally when the CBM treatment was performed with the deactivated catalyst (Table S4†). Consequently, –SO_3_H groups must play a prominent role in catalysing the formation of soluble reaction products during the CBM treatment or the subsequent hydrolysis process.

Solubility tests performed with the CBM treated samples revealed that mechanocatalytic depolymerization only occurred when the active sulfonated carbon catalyst was used during the treatment. When the CBM treatment was conducted with the non-sulfonated or the deactivated materials the solubilities were 0.7% and 1.9%, respectively (Table S4†). In contrast, 32% of the sample CBM-7/2 was soluble in Millipore water at room temperature. The soluble fraction was composed of oligosaccharides (95%) with a DP up to 10 (Fig. S8C†) with additional minor amounts of monosaccharides (4%) and dehydration products (1%) formed. Based on the finding that individually ball-milled cellulose or cellulose subjected to CBM treatment with the non-sulfonated or deactivated carbon displayed only marginal solubility it is concluded that the enhanced solubility can be attributed to a mechanochemical depolymerization catalysed by –SO_3_H groups. It is further noteworthy, that unlike the soluble fraction from ball-milled cellulose, the sample CBM-7/2 contained dehydration products. This can be interpreted as a further indication that the CBM treatment induces sufficient proximity between the catalyst and reaction products to facilitate a reaction.

### Stability of the sulfonated carbon catalyst

The CBM treatment and the subsequent hydrolysis process expose the sulfonated carbon catalyst to harsh mechanical forces and hydrothermal conditions. Thus, the catalyst may be subject to deactivation by attrition or –SO_3_H leaching. The stability of the catalyst was therefore studied during repeated CBM treatment & hydrolysis cycles. The S/C ratio was chosen such as to achieve levels of conversion far from 100%.

**Fig. 10:**
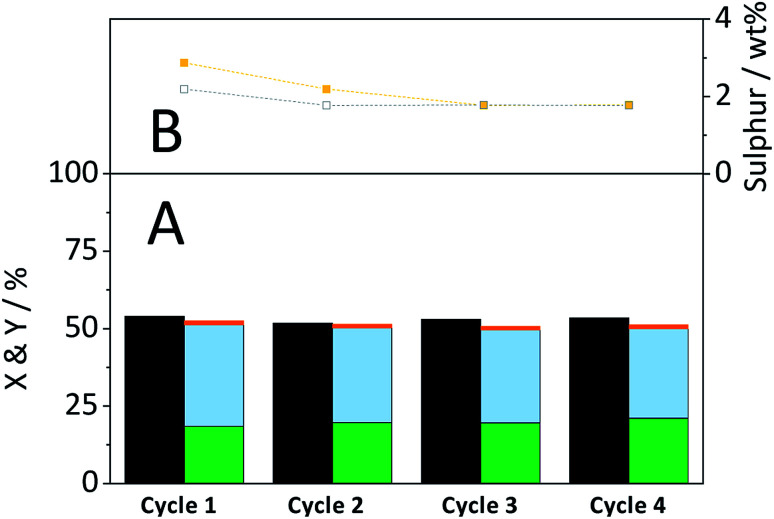
(A) Conversion (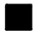) and yields of oligosaccharides (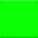), monosaccharides (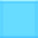) and dehydration products (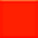) during recyclability experiments. (B) Sulphur content of catalyst before (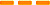) and after (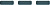) a reaction cycle. Reaction conditions: 165 °C, 6.5 h, 3 wt% CBM-7/2, 1 mM EDTA & 15-C-5, *τ* = 35 min, impeller speed = 600 rpm.

Fig. 10A shows the conversion and yields achieved during four consecutive CBM-hydrolysis cycles. After each cycle, the cellulose-catalyst mixture was recovered and the former was removed through dissolution in a 72% H_2_SO_4_ solution. The recycled catalyst and fresh cellulose were then subjected to repeated CBM treatment and hydrolysis in the semi-batch reactor. During the first cycle a minor increase/decrease in the selectivity of forming oligosaccharides/monosaccharides occurred, which can be attributed to the leaching of –SO_3_H groups (Fig. 10B). The density of active sites decreased from 869 μmol g^−1^ in the fresh catalyst to 556 μmol g^−1^ during the first two cycles. Importantly, the remaining sulfonic acid groups of the catalyst proved to be stable during the third and fourth cycle. The chemical identity of the remaining sulphur in the catalyst was confirmed by recording FTIR spectra of the spent materials (Fig. S13B†). The FTIR spectra of the spent materials show an absorption band at 1040 cm^−1^, previously assigned to the symmetric stretching mode of O=S=O in –SO_3_H groups.

Despite leaching of –SO_3_H groups, the catalytic activity remained stable over the four cycles. Consequently, leached sulphur containing species did not contribute to product formation during the CBM treatment or during hydrolysis in the semi-batch.

It is also important to point out that a part of the apparently reduced –SO_3_H density is not due to the leaching of sulphur from the material but is rather caused by changes in the elemental composition of the catalyst. Individual ball-milling of the catalyst led to an increase in the oxygen content (fresh: 31.8%; ball-milled: 37.5%) which consequently also reduced the sulfonic acid group density (869 to 788 μmol g^−1^). Besides leaching, the catalyst also underwent a reduction in its particle size during the recyclability experiments. SEM images of the recovered catalyst (Fig. S13A†) show that ball-milling led to a reduction in size and changed the morphology of the particles. The median of the particle size distribution after the first cycle was reduced to 16.7 μm. During the second and third cycle the median decreased further to 13.8 and 13.3 μm, respectively.

### Influence of the substrate-to-catalyst ratio during mechanocatalytic depolymerization

**Fig. 11:**
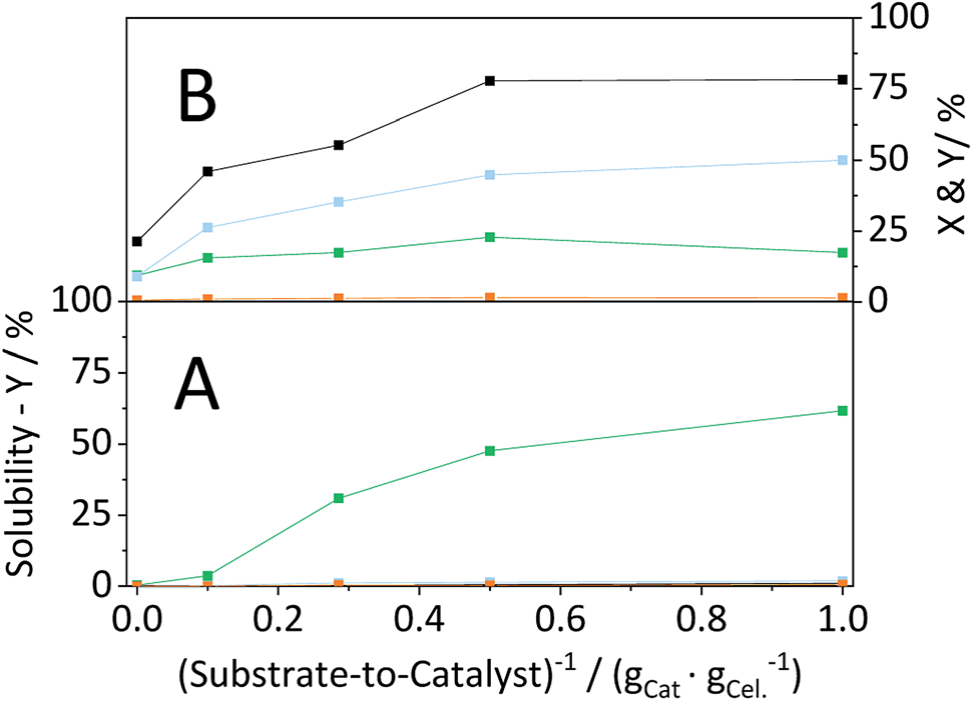
Conversion (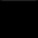) and yields of oligosaccharides (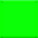), monosaccharides (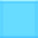) and dehydration products (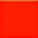) achieved with CBM treated cellulose as a function of the substrate-to-catalyst ratio, during (A) solubility tests (25 °C, 1 g CBM-cellulose per 10 mL Millipore water, 1 h) and (B) hydrolysis in the semi-batch reactor (165 °C, 6.5 h, 3 wt% CBM-cellulose, 1 mM EDTA & 15-C-5, *τ* = 35 min, impeller speed = 600 rpm).

Having established that the catalyst was stable, in a next step efforts were made to optimize the conversion of the substrate. To this end, we investigated the influence of the S/C ratio on the mechanocatalytic depolymerization and the hydrolysis in the semi-batch reactor. Regardless of the S/C ratio, CBM treatment led to extensive amorphization, whereas higher catalyst loadings resulted in slightly lower CI's (Table S4†). However, the S/C ratio directly defined the amount of soluble oligosaccharides formed during CBM treatment. Whereas individually ball-milled cellulose was only 0.4% soluble, the sample CBM-10/1 exhibited a tenfold higher solubility (3.9%). The solubility of CBM treated cellulose increased further with the inverse of the S/C ratio and reached 64.1% for CBM-1/1 treated cellulose (Fig. 11A).

Following CBM treatment at varying S/C ratios, the samples were hydrolysed in the semi-batch reactor (Fig. 11B). During hydrolysis in the semi-batch reactor the oligosaccharides formed during CBM treatment were increasingly converted to monosaccharides at higher S/C ratios. At a S/C ratio of 1/1, 78.2% of cellulose was converted to yield 17.4%, 50.0% and 1.4% oligosaccharides, monosaccharides and dehydration products, respectively.

### Hydrolysis of softwood

**Fig. 12:**
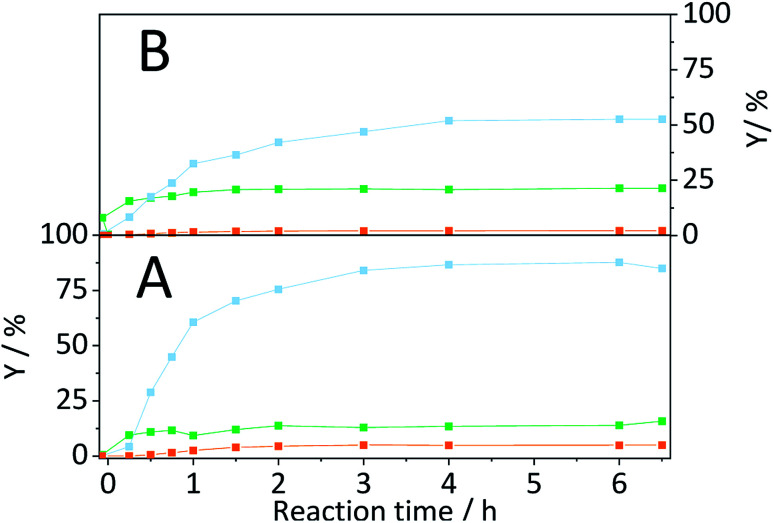
Yields of (A) C_5_- and (B) C_6_-derived oligosaccharides (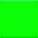), monosaccharides (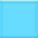) and dehydration products (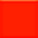) achieved with CBM-1/1 treated softwood. Reaction conditions: 165 °C, 6.5 h, 2 wt% CBM-1/1 softwood, 1 mM EDTA & 15-C-5, *τ* = 35 min, impeller speed = 600 rpm.

To assess the applicability of the developed conversion strategy to real lignocellulose, in a final step the hydrolysis of spruce fir wood (*picae abies*) was investigated. Compositional analysis^48,53,54^ revealed that the spruce fir wood was composed of hemicellulose (20.5%), cellulose (43.3%) and lignin (32.3%) as well as minor amounts of extractives (3.2%) and ash (0.5%). Both hemicellulose and cellulose undergo acid catalysed hydrolysis following very similar chemical pathways. Like C_6_-based compounds C_5_-containing oligosaccharides undergo hydrolysis to form the corresponding monosaccharides that can subsequently undergo acid catalysed dehydration to furfural. However, unlike cellulose, the hemicellulose in the spruce wood is a branched heteropolymer composed of mannose (10.7%), xylose (7.7%) and arabinose (2.1%) units. The branching of the backbone hinders the aggregation of the polymer chains and thereby prevents the formation of inter-chain H-bonds. Consequently, hemicellulose exhibits a significantly higher tendency to undergo auto-^8^ or acid catalysed^14^ hydrolysis. In contrast to the cellulosic constituents, lignin is a crosslinked heteropolymer composed of phenyl-propanoid building blocks interconnected through different carbon–oxygen and carbon–carbon bonds. The presence of carbon–carbon bonds implies that lignin cannot be fully depolymerized using only Brønsted acids under the conditions employed in this study. Fig. 12 shows the yields for C_5_- and C_6_-derived reaction products achieved with CBM-treated spruce wood (S/C = 1/1) during hydrolysis in the semi-batch reactor. As evidenced by the higher yields, the C_5_-containing hemicellulose was significantly more reactive. The corresponding yields for C_5_ (xylose and arabinose) derived oligosaccharides, monosaccharides and dehydration products were 15.7%, 84.9% and 4.9%, respectively.

The yields in C_6_ (glucose and mannose) derived oligosaccharides, monosaccharides and dehydration products were 21.4%, 52.6% and 2.2%, respectively. As expected, lignin did not undergo extensive depolymerization. Only traces of the lignin depolymerization markers vanillin, 2-methoxy-4-propyl phenol and 4-acetoxy-3-methoxy acetophenone could be identified in the reactor effluent. Solubility tests further showed that the CBM-treated softwood underwent mechanocatalytic hydrolysis to yield 8.2% and 6.1%, C_5_- and C_6_-derived compounds, respectively. The high degree of depolymerization of both cellulose and hemicellulose confirm that the developed methodology can be extrapolated to real lignocellulose. Furthermore, the achieved yields in C_5_- and C_6_-derived products are the highest ever reported in literature for a continuous hydrolysis process that does not rely on the use of strong homogeneous Brønsted acids. Remarkably, the yields even show that the developed conversion strategy can compete with those achieved with conventional diluted homogeneous acid processes (C_5_: 88%, C_6_: 50–60%).^12–14^

## Conclusions

A two-step process composed of mechanocatalytic pre-treatment and secondary hydrolysis in a semi-batch reactor was developed using sulfonated carbons as recyclable solid acid catalyst. The solid acid catalysed hydrolysis of pristine cellulose is shown to be strongly inhibited by the substrate's insolubility. In this case, the formation of soluble oligosaccharides occurs to a large extent through a homogeneously catalysed cleavage of glycosidic bonds (auto-hydrolysis, hydrolysis by EDTA and by leached species from the catalyst). Auto-hydrolysis is shown to preferentially occur from less ordered structural domains, which rationalized the finding that amorphized cellulose exhibited a higher specific reactivity when converted in presence of solid acids. When ball-milling is performed in presence of the sulfonated carbon catalyst (concerted ball-milling, CBM), mechanocatalytic depolymerization leads to the formation of up to 64% soluble oligosaccharides. Secondary hydrolysis of the mechanocatalytically pre-treated cellulose in the semi-batch reactor was no longer restrained by the substrate's insolubility. CBM treatment with carbon materials featuring different surface chemistries revealed the crucial role of strong Brønsted acid sites in facilitating a mechanocatalytic hydrolysis. This finding provides valuable insights into the structural properties of effective solid acids and can guide the future design of catalysts for this application. The hydrothermal and mechanical stability of the catalyst coupled with the very high yields achieved during hydrolysis of softwood demonstrated that the developed conversion strategy offers a real perspective to overcome major limitations of conventional hydrolysis processes. To further enhance its applicability to the conversion of biomass on a large scale, future process intensification studies will aim at increasing the semi-batch reactor's productivity and at facilitating complete conversion of oligo- to monosaccharides.

## Conflicts of interest

The authors declare no conflict of interest.

## Supplementary Material

RA-009-C9RA07668A-s001
