# Supplementary material for: Mechanochemistry-assisted hydrolysis of softwood over stable sulfonated carbon catalysts in a semi-batch process
Source: RSC Adv. 2019 Oct 18;9(57):33525–38. doi: 10.1039/c9ra07668a (PMC9073368; doi:10.1039/c9ra07668a)
Supplement: RA-009-C9RA07668A-s001 [file RA-009-C9RA07668A-s001.pdf]

Electronic Supplementary Material (ESI) for RSC Advances.
This journal is © The Royal Society of Chemistry 2019

Supplementary Material: Mechanochemistry-Assisted Hydrolysis of Softwood over Stable Sulfonated Carbon Catalysts in a Semi-Batch Process

David Scholz, Jingwei Xie, Oliver Kröcher and Frédéric Vogel

1. Physicochemical characterization of catalyst

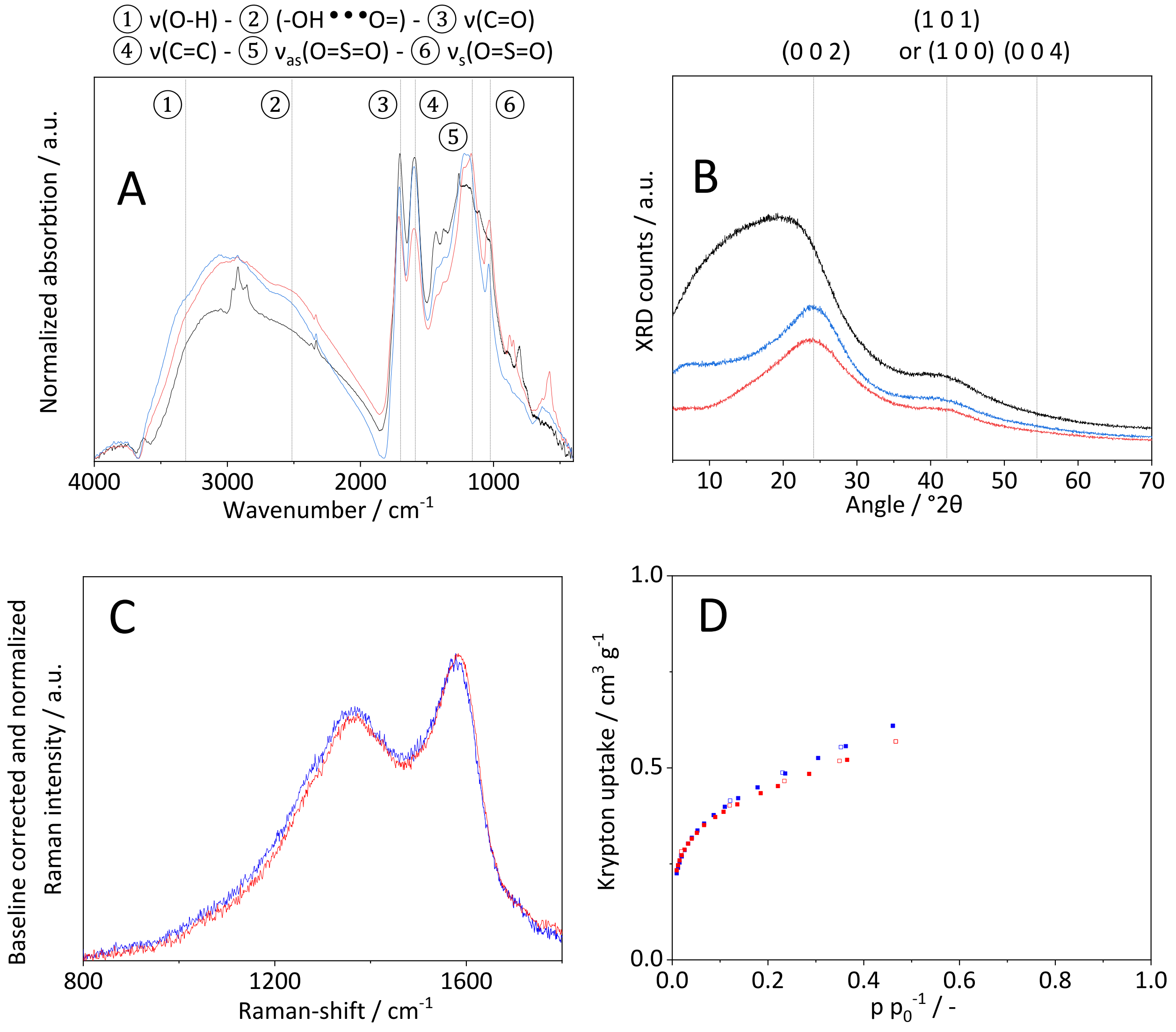

Figure 1. (A) FTIR spectra of carbonized (−), sulfonated (−) and hydrothermally treated (−) catalyst samples. **(B)** XRD patterns of carbonized (−), sulfonated (−) and hydrothermally treated (−) catalyst samples. **(C)** Raman spectra of sulfonated (−) and hydrothermally treated (−) catalyst samples. **(D)** Krypton adsorption/desorption isotherms of sulfonated (■/□) and hydrothermally treated (■/□) catalyst samples.

2. Physicochemical properties of catalyst

Table 1. Physicochemical properties of catalyst samples. [a] Determined using elemental analysis. [b] Calculated from the sulphur content. [c] Determined using Krypton physisorption. [d] Determined using laser diffraction. BM denotes ball-milled.

Sample	C [%] [a]	H [%] [a]	O [%] [a]	$-SO_3H$ [μmol g^{-1}] [b]	BET Area [m^2 g^{-1}] [c]	Particle size [μm][d]
C-350	74.9±0.2	3.9±0.1	21.1±0.1	0	n.A.	56.4
C-350-SO_3H	54.5±1.1	3.5±0.2	36.5±0.2	2068±57	2.1	52.8
C-350-SO_3H-HT	63.2±1.1	3.4±0.1	31.8±1.0	869±29	2.2	27.6
C-350-SO_3H-HT-BM	57.0	2.9	37.5	788	n.A.	12.5

3. Yields, temperature profile and flow-rate during hydrolysis of cellulose over C-350-SO_3H-HT

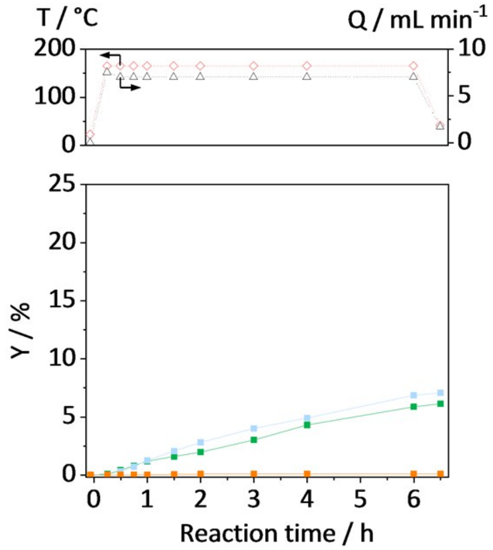

Figure 2: Yields of oligosaccharides (■), monosaccharides (■) and dehydration products (■) achieved with pristine cellulose in presence of C-350-SO_3H-HT. Reaction conditions: 165 °C, 6.5 h, 3 wt% cellulose, S/C = 7/2, 1 mM EDTA & 15-C-5, τ = 35 min., impeller speed = 600 rpm.

4. Influence of the impeller speed on the conversion of cellulose in presence of C-350-SO_3H-HT

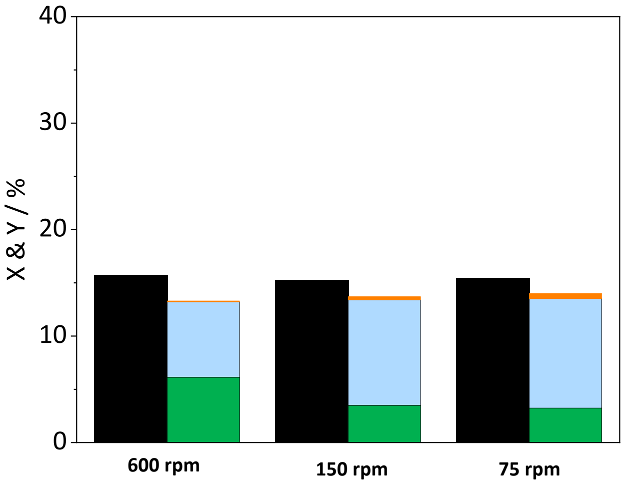

Figure 3: Conversion (■) and yields of oligosaccharides (■), monosaccharides (■) and dehydration products (■) achieved during the hydrolysis of cellulose in presence of EDTA and 350-SO_3H-HT at different impeller speeds. Reaction conditions: 165°C, 6.5 h, 3 wt% cellulose, S/C = 7/2, 1 mM EDTA & 15-C-5, τ = 35 minutes.

5. Full width at half maximum (FWHM) of deconvoluted diffraction peaks

Table 2. FWHM of the deconvoluted diffraction peaks determined for the crystal planes in cellulose.

Sample	FWHM (1 0 1) / °	FWHM (1 0 $\bar{1}$) / °	FWHM (0 2 1) / °	FWHM (0 0 2) / °	FWHM (0 0 4) / °
Cellulose	2.43	2.04	1.42	1.46	1.88
BM-1 h	2.28	2.04	2.74	1.47	1.73
BM-4 h	2.68	3.42	3.14	1.41	1.70
BM-8 h	2.87	4.36	4.40	1.39	1.72
BM-18 h	3.54	4.98	4.20	1.27	1.74
BM-54 h	3.52	6.59	4.85	1.23	2.46

6. Properties of pre-treated cellulose samples

Table 3. Properties, conversion and reaction yields achieved with pre-treated cellulose in presence of C-350-SO_3H-HT. Reaction conditions: 165 °C, 6.5 h, 3 wt% pre-treated cellulose, S/C = 7/2, 1 mM EDTA & 15-C-5, τ = 35 min., impeller speed = 600 rpm. * O: Oligosaccharides, M: Monosaccharides and D: Dehydration products. BM denotes ball-milled.

Sample	Conversion / %	Yield: O-M-D*/ %	Particle size ($d_{50,V}$) / µm	Crystallite size /nm	CI / %	Solubility – yield / %
Cellulose	15.2	6.1/7.1/0.1	50.8	4.8	65.3	0.05 ± 0.01
BM-1 h	16.6	2.5/9.7/0.4	22.4	4.3	64.7	0.19 ± 0.01
BM-4 h	17.3	4.8/11.3/0.4	20.5	3.9	59.2	0.27 ± 0.01
BM-8 h	22.5	6.1/13.6/0.7	21.8	3.6	59.1	0.32 ± 0.02
BM-18 h	27.2	7.8/15.9/0.8	24.6	3.5	57.4	0.41 ± 0.01
BM-54 h	32.9	9.2/19.5/0.9	22.4	3.5	56.5	0.79 ± 0.02
BM-18 h + 5% H_2O	34.0	12.0/19.4/0.9	43.4	3.4	52.0	0.53 ± 0.01
Amorphous cellulose	27.1	14.8/10.4/0.6	93.3	2.2	20.1	0.95 ± 0.36

7. Crystallinity index, particle size and SEM images of ball-milled cellulose

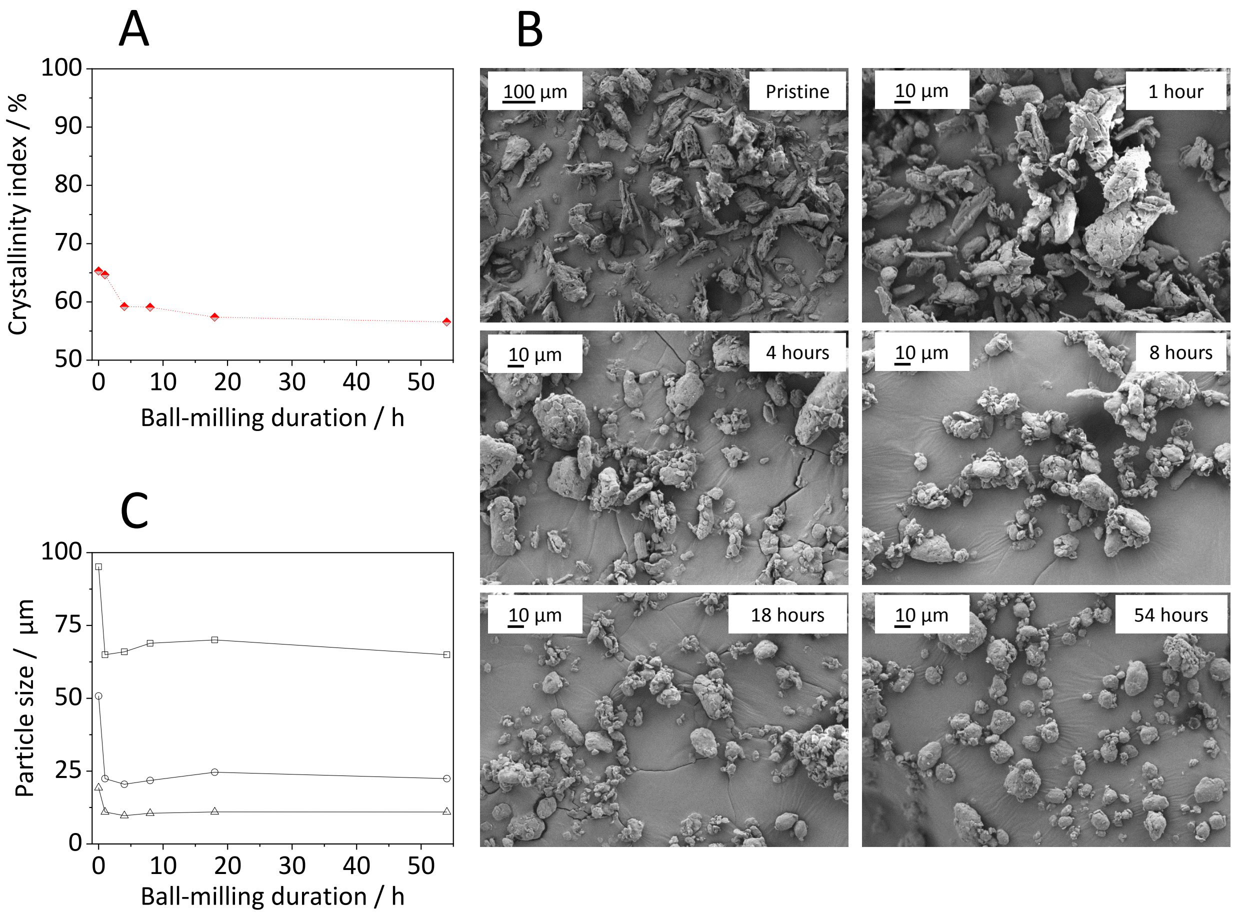

Figure 4. (A) Crystallinity index (◆), **(B)** SEM images and **(C)** particle size distribution ($d_{10,V}$: △ – $d_{50,V}$: ○– $d_{90,V}$: □) of ball-milled cellulose.

8. Crystallinity index, particle size and SEM images of wet ball-milled cellulose

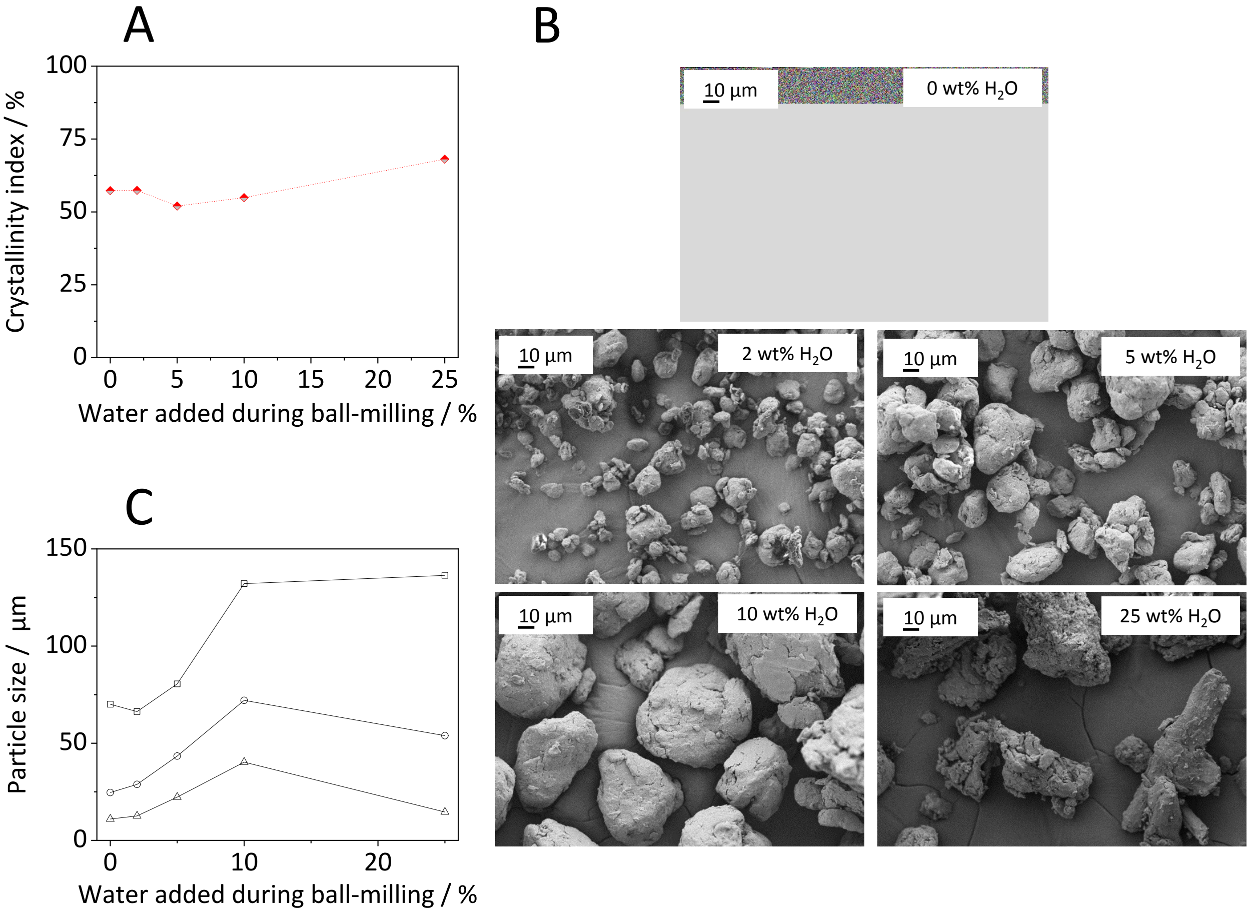

Figure 5. (A) Crystallinity index (◆), **(B)** SEM images and **(C)** particle size distribution ($d_{10,V}$: △ – $d_{50,V}$: ○– $d_{90,V}$: □) of cellulose ball-milled in presence of H_2O.

9. Yields during hydrolysis of wet ball-milled cellulose in presence of C-350-SO_3H-HT

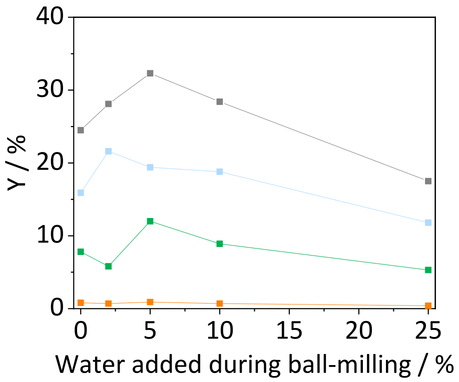

Figure 6. Yields of oligosaccharides (■), monosaccharides (■), dehydration products (■) and total yield (■) achieved with wet ball-milled cellulose in presence of C-350-SO_3H-HT. Reaction conditions: 165 °C, 6.5 h, 3 wt% wet ball-milled cellulose, S/C = 7/2, 1 mM EDTA & 15-C-5, τ = 35 min., impeller speed = 600 rpm.

10. FTIR Spectra of pre-treated cellulose samples

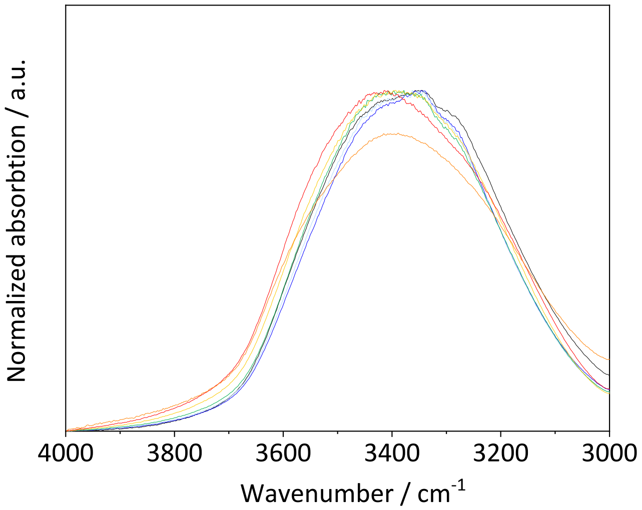

Figure 7. FTIR spectra of pristine (–), BM-1 h (–), BM-4 h (–), BM-8 h (–), BM-18 h (–), BM-54 h (–), BM-18 h + 5% H_2O (–) and amorphous cellulose (–) in the 4000-3000 cm^{-1} wavenumber region.

11. Ion-chromatograms of soluble species in pristine and pre-treated cellulose

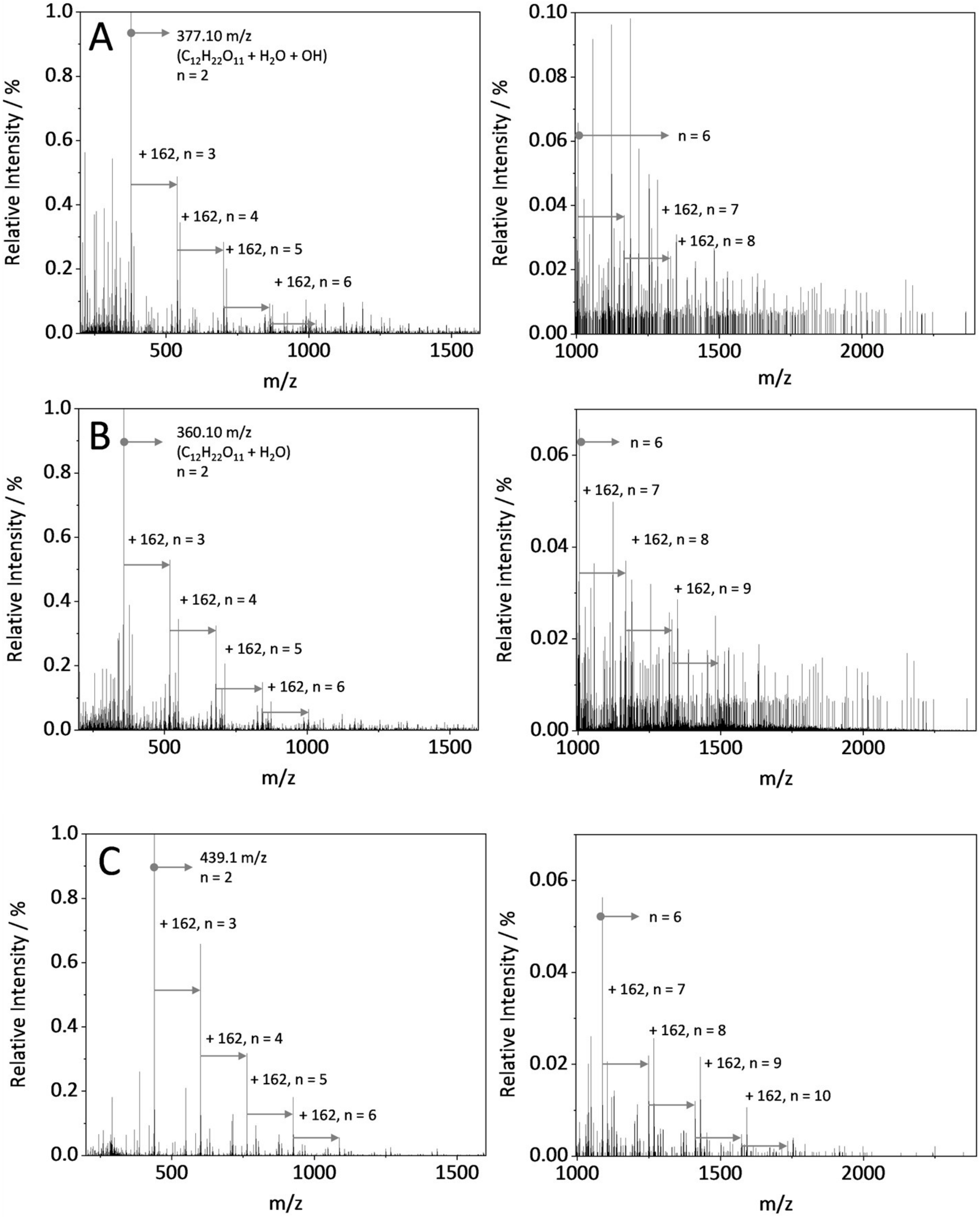

Figure 8. ESI-MS total ion-chromatograms of soluble species in **(A)** Cellulose, **(B)** BM-54 h and **(C)** CBM-10/1.

12. XRD patterns of cellulose residues recovered after pathway experiments

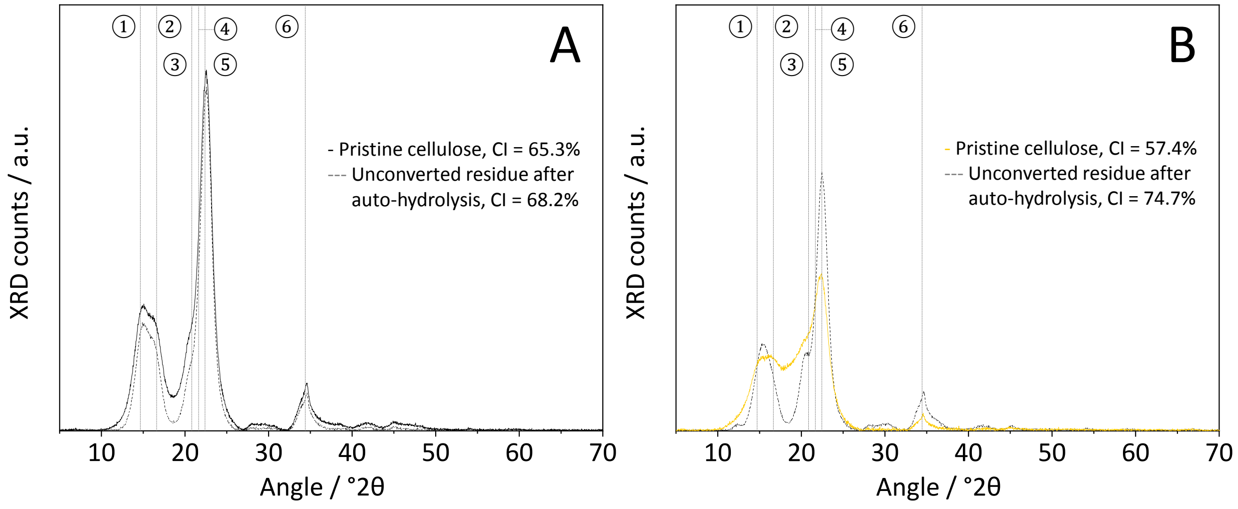

Figure 9. (A) Powder X-ray diffraction patterns of pristine cellulose (–) and the residue recovered after the auto-hydrolysis experiment (---). **(B)** Powder X-ray diffraction patterns of cellulose ball-milled for 18 hours (–) and the residue recovered after the auto-hydrolysis experiment (---).① - (1 0 1), ② - (1 0 $\bar{1}$), ③ - (0 2 1), ④ - para-crystalline, ⑤ - (0 0 2), and ⑥ - (0 0 4).

13. Measured and calculated XRD pattern of a physical mixture of ball-milled cellulose and ball-milled C-350-SO_3H-HT

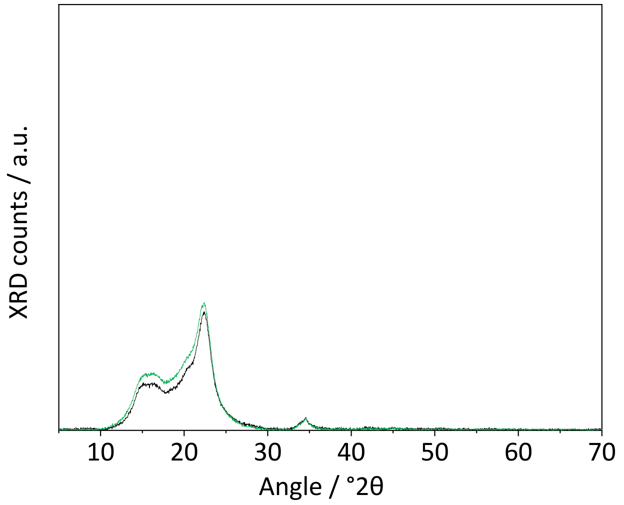

Figure 10. XRD diffraction patterns of a physical mixture (S/C = 7/2) of individually ball-milled cellulose and ball-milled C-350-SO_3H-HT (–) and the calculated diffraction pattern of a mixture (S/C = 7/2) of ball-milled cellulose and ball-milled C-350-SO_3H-HT determined by linear combination (–).

14. Liquid phase adsorption of cellobiose onto C-350-SO_3H-HT

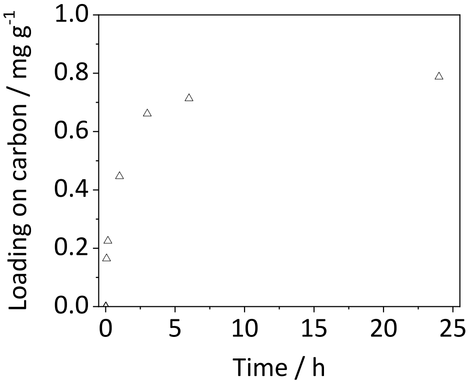

Figure 11. Cellobiose adsorption onto C-350-SO_3H-HT. Conditions: 25 °C, 100 mg C-350-SO_3H-HT per 1 mL of 20 mM cellobiose solution.

15. Particle size distribution of the sample CBM -7/2

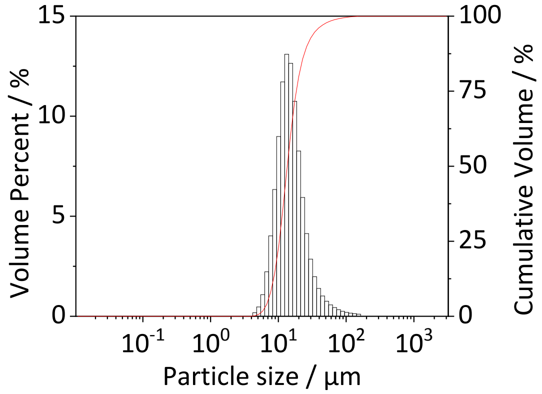

Figure 12. Particle size distribution of the sample CBM-7/2 measured with laser diffraction.

16. Properties of CBM treated cellulose

Table 4. Properties, conversion and reaction yields achieved with CBM treated cellulose. Reaction conditions: 165 °C, 6.5 h, 3 wt% CBM cellulose, 1 mM EDTA & 15-C-5, τ = 35 min., impeller speed = 600 rpm. *O: Oligosaccharides, M: Monosaccharides and D: Dehydration products. CBM denotes concerted ball-milling.

Sample	Conversion / %	Product yield: O-M-D*/ %	Particle size ($d_{50,V}$) / µm	CI / %	Solubility – yield / %
CBM-10/1	21.3	9.4/9/0.6	19.1	36.2	3.9
CBM-7/2	55.3	17.4/35.3/1.2	13.5	31.6	32.4
CBM-2/1	77.8	22.8/44.8/1.5	14.9	28.9	49.2
CBM-1/1	78.2	17.4/50.0/1.4	15.0	24.8	64.1
CBM-7/2 - No sulf.	26.9	12.8/12.3/0.3	7.6	35.9	0.7 ± 0.1
CBM-7/2 - Deac.	35.3	15.4/18.5/0.2	9.9	34.7	1.9 ± 0.4

17. SEM Images and FTIR spectra of fresh and recycled catalyst

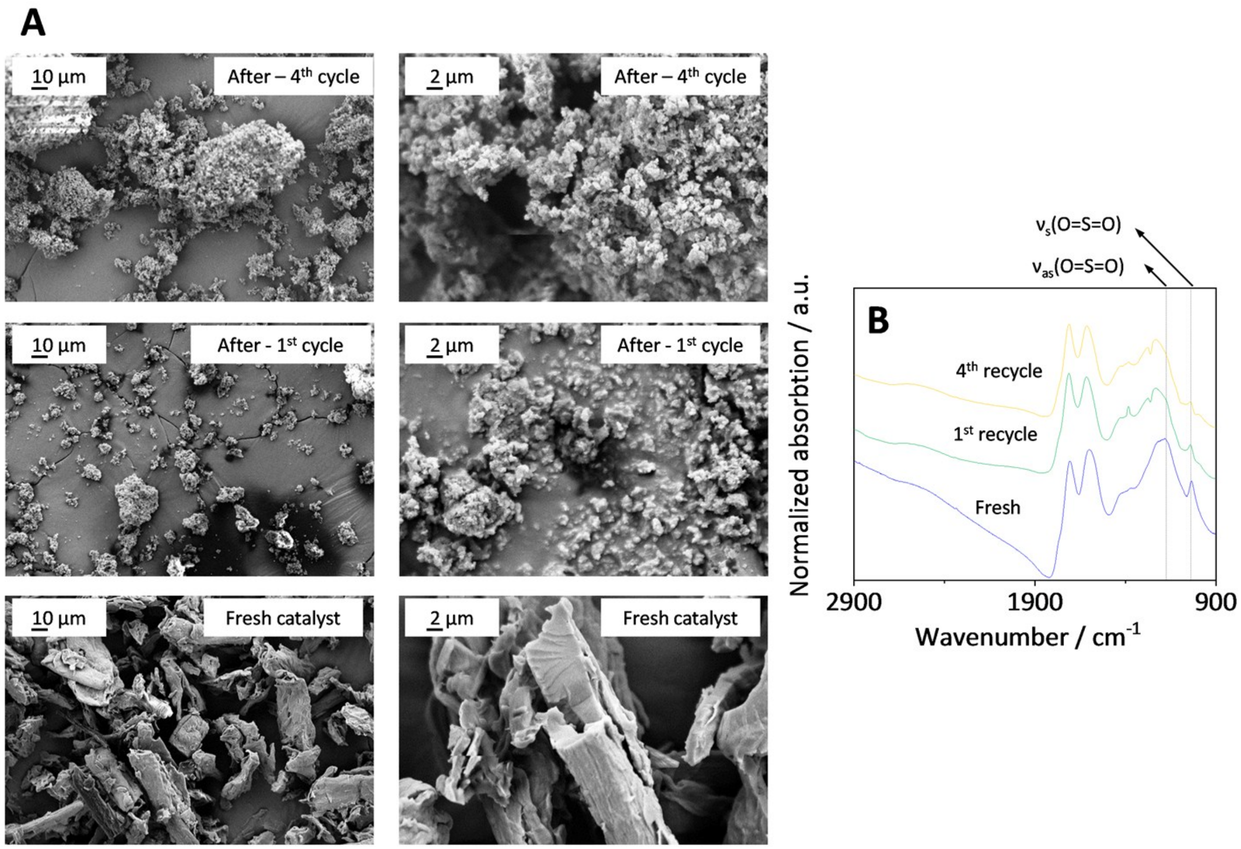

Figure 13. (A) SEM images and (**B)** FTIR spectra of fresh and recycled catalyst.

18. XRD diffraction patterns of CBM treated cellulose prepared at different substrate-to-catalyst ratios

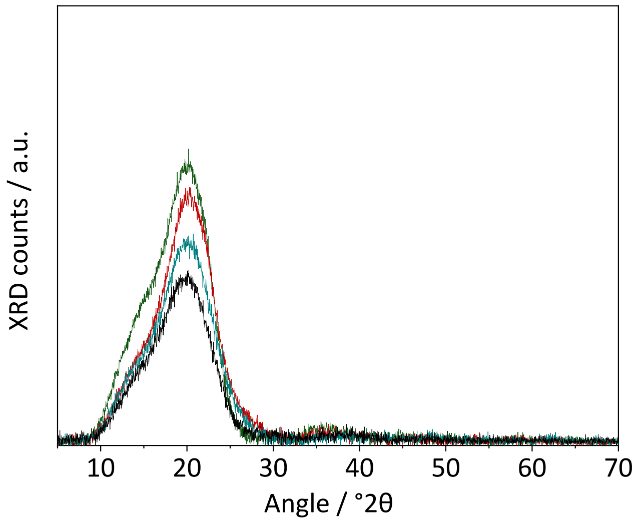

Figure 14. (A) Powder X-ray diffraction patterns of cellulose subjected to CBM treatment at different substrate-to-catalyst ratios, CBM-10/1 (–), CBM-7/2 (–), CBM-2/1 (–) and CBM-1/1 (–).
